# Approaching Scarless Wound Healing: From Passive Anti‐Fibrotic to Proactive and Programmable Pro‐Regenerative Strategies

**DOI:** 10.1002/advs.202521824

**Published:** 2026-02-24

**Authors:** Meimei Fu, Zhuoyi Huang, Jintao Li, Yue Li, Zhou Fang, Yiwen Jiang, Keke Wu, Jinshan Guo

**Affiliations:** ^1^ Department of Histology and Embryology NMPA Key Laboratory for Safety Evaluation of Cosmetics GDMPA Key Laboratory of Key Technologies for Cosmetics Safety and Efficacy Evaluation School of Basic Medical Sciences Southern Medical University Guangzhou P. R. China; ^2^ Suzhou Institute of Biomedical Engineering and Technology Chinese Academy of Sciences Suzhou P. R. China; ^3^ Department of Sports Medicine Center for Orthopedic Surgery, Orthopedic Hospital of Guangdong Province, The Third School of Clinical Medicine, Guangdong Provincial Key Laboratory of Bone and Joint Degeneration Diseases The Third Affiliated Hospital of Southern Medical University Guangzhou P. R. China; ^4^ Department of Plastic and Aesthetic Surgery Nanfang Hospital of Southern Medical University Guangzhou Guangdong Province P. R. China; ^5^ School of Materials Science and Engineering Changchun University of Science and Technology Changchun P. R. China; ^6^ School of Biomedical Engineering, Affiliated Cancer Hospital & Institute Guangzhou Medical University Guangzhou P. R. China

**Keywords:** anti‐fibrotic, pro‐regenerative, scarless wound healing, smart material

## Abstract

Pathological scarring after skin wound healing poses a substantial clinical difficulty, essentially representing a fibrotic outcome of dysregulated tissue repair. This review provides a comprehensive analysis of the physiological processes of wound healing and key factors in scar formation, focusing on the unique mechanisms of scarless regeneration models such as early‐stage fetuses and spiny mouse (*Acomys cahirinus*). These models rely heavily on the immune microenvironment's regulation, the dynamic remodeling of the extracellular matrix (ECM), and precise cell behavior regulation to achieve perfect regeneration. Building upon this foundation, this review delves into emerging scar prevention methods from the perspective of tissue engineering materials. These encompass multi‐dimensional interventions, including the construction of biomimetic microenvironments, regulation of key cell fates, sequential multi‐target intervention in pathological processes, and the integration of smart materials with advanced manufacturing technologies. Through interdisciplinary integration and innovation, shifting scar prevention and treatment strategies from passive anti‐fibrosis to proactive promoting regeneration, guiding wounds toward true structural and functional regeneration, emerges as a promising path to overcome current limitations in scar management.

## Graphical abstract

This review outlines the wound healing process and factors influencing scar formation, explores how certain animals and human fetuses achieve scarless skin or tissue regeneration, and details tissue engineering material strategies employed in scarless wound dressings. These advanced therapy strategies aim to actively guide wound repair toward true regeneration, proactively restoring healthy and fully functional skin tissue.

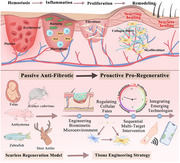

## 1 Introduction

The skin, the largest organ of the body, not only functions as a crucial physical barrier against the invasion of external pathogens, chemicals, and harmful environmental agents, but also actively participates in thermoregulation and sensory perception [1, 2]. The failure to promptly heal a skin wound with a compromised barrier function predisposes individuals to deep tissue infections, with the potential for progression to life‐threatening systemic infections, including sepsis [3, 4]. Wound healing is a programmed dynamic process initiated by hemostasis, involving multiple stages such as platelet activation, inflammatory cell infiltration, angiogenesis, and tissue remodeling [5, 6, 7]. Ultimately, the nascent skin tissue undergoes a maturation process, transforming into functional scar tissue or regaining its structural integrity [8].

Scar formation presents a considerable clinical challenge after skin injury, essentially a pathological outcome driven by a dysregulated healing process [9]. Scar formation is closely associated with multiple factors, including an imbalance in the inflammatory response, abnormal cellular function, and disrupted ECM metabolism [10, 11]. Normal skin and scar tissue differ significantly in their molecular expression, cellular makeup, and structural organization, which collectively form the pathological foundation of scarring [12, 13, 14]. Current interventions for established scars employ a variety of approaches, primarily involving laser therapy, radiation therapy, cryosurgery, silicone dressings, compression therapy, and local injections of medications such as corticosteroids and 5‐fluorouracil [15, 16, 17]. Despite their primary goal of enhancing cosmetic appearance and alleviating symptoms, these approaches are often hampered by limitations such as significant inter‐individual variability, lengthy treatment cycles, high recurrence rates, and insufficient correction of deep tissue contractures, all of which contribute to an overall unsatisfactory therapeutic outcome [18, 19, 20]. Given these limitations, the scar teatment strategy is currently undergoing a paradigm shift from a corrective approach targeting mature scars to a preemptive one approaching scarless wound healing [9, 21]. This strategy aims to intervene at critical stages of the healing process, preventing scar formation through multi‐level regulation, thereby shifting the treatment approach from passive management to proactive prevention.

**FIGURE 1:**
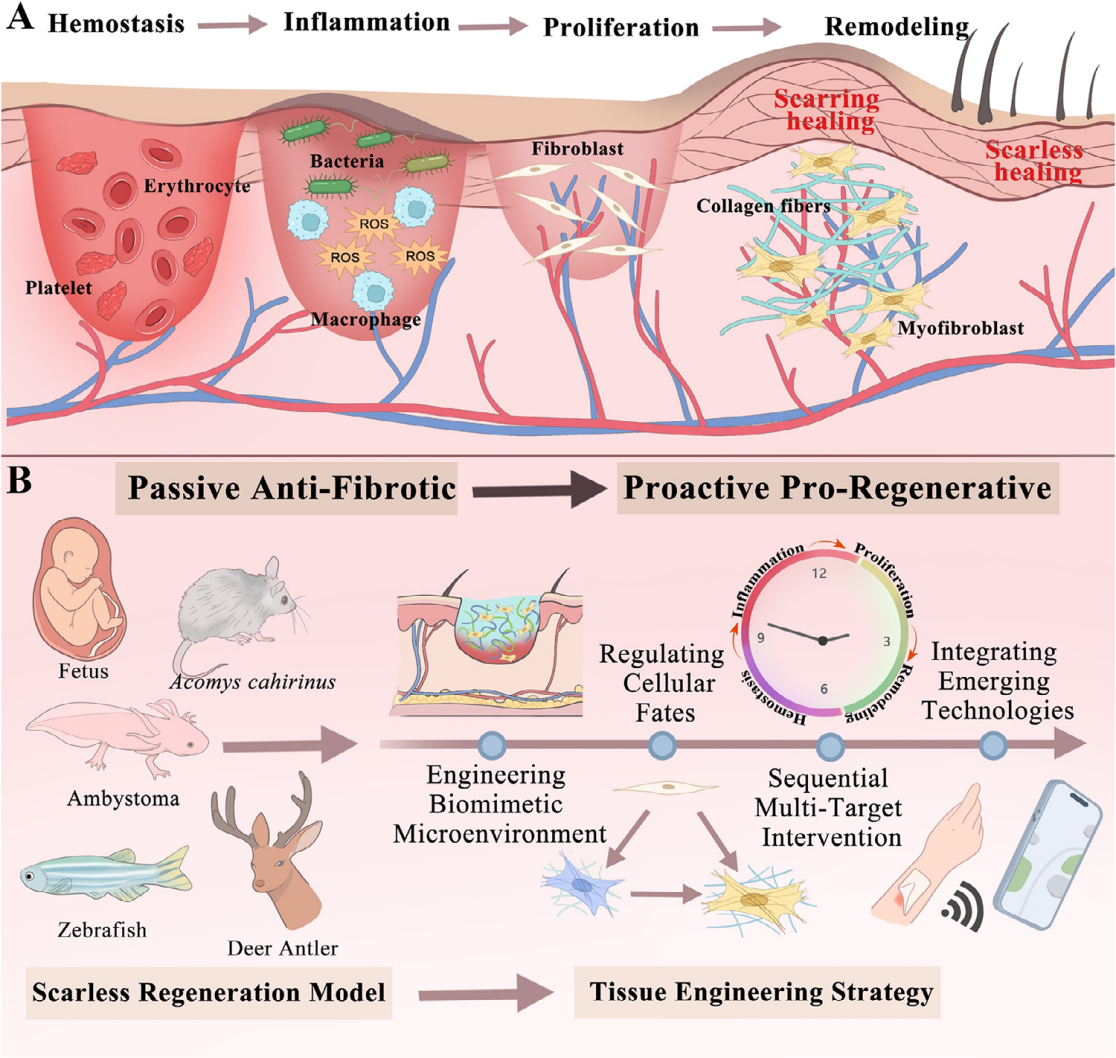
Guiding scarless wound healing: from passive anti‐fibrotic response to programmable pro‐regenerative strategy. (A) The physiological process of wound healing and key factors in scar formation. (B) Scarless regeneration models and tissue engineering material strategies.

This review seeks to chart a transformative pathway from passive scar intervention to active functional regeneration. This will be achieved by systematically analyzing the core mechanisms of wound healing, summarizing the defining features of scarless regeneration models, and exploring tissue engineering strategies. These strategies involve the construction of biomimetic microenvironments, the regulation of cell fate, sequential multi‐target interventions, and the integration of emerging technologies (Figure 1).

## 2 The Healing Process of Skin Wounds and Factors Affecting Scar Formation

**FIGURE 2:**
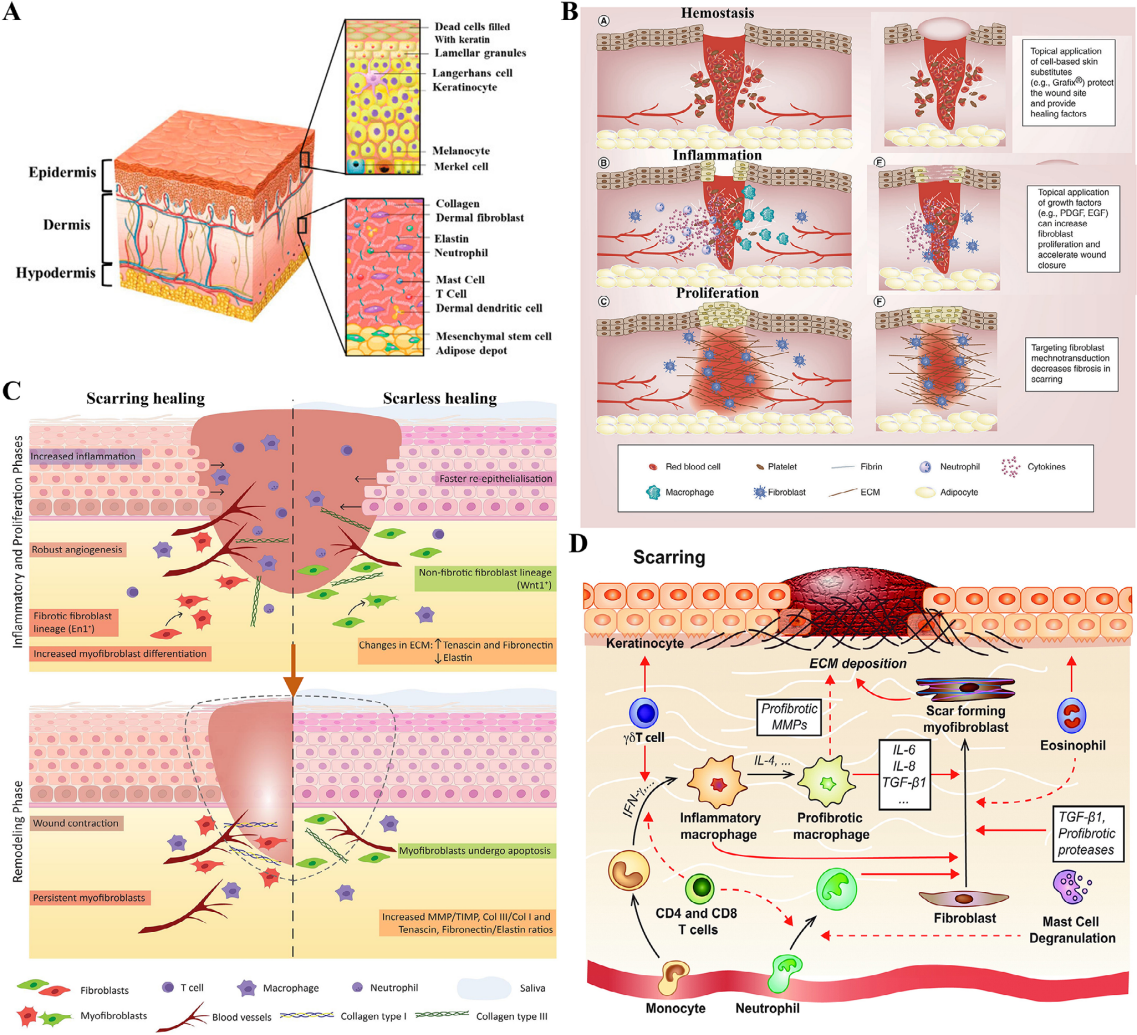
The healing process of skin wounds and scar formation. (A) Schematic illustration of human skin structure. Reproduced under terms of the CC‐BY license [24]. Copyright 2020, Tavakoli S, et al., published by MDPI. (B) Wound healing: hemostasis, inflammation, and proliferation stages and treatment strategies. Reproduced with permission [38]. Copyright 2018, Taylor & Francis. (C) Molecular behavior and cellular events in scarring healing and scarless healing. Reproduced under terms of the CC‐BY license [63]. Copyright 2021, Pereira D and Sequeira I. (D) Molecular and cellular mechanisms involved in scar formation. Reproduced with permission [76]. Copyright 2018, Mary Ann Liebert.

The skin is composed of three consecutive layers, from superficial to deep: the epidermis, dermis, and hypodermis, collectively forming a critical physical barrier (Figure 2A) [22, 23, 24]. The epidermis, the skin's outermost barrier, generates a resilient stratum corneum via the proliferation, differentiation, and final desquamation of keratinocytes, thereby executing its key functions of preventing transepidermal water loss and resisting pathogen invasion [25, 26]. Scattered within it are melanocytes and Langerhans cells, with distinct roles in UV protection and immune surveillance, respectively [27, 28]. The dermis lies beneath the epidermis and is a dense connective tissue rich in type I and III collagen and elastic fibers, which collectively form the mechanical foundation for the skin's strength, toughness, and elasticity [29]. This layer also contains critical structures such as nerves, blood vessels, hair follicles, sebaceous glands, and sweat glands, which facilitate sensory transduction, thermoregulation, and secretion/excretion, respectively [30]. The hypodermis, the skin's deepest layer, is primarily comprised of adipocytes, thereby performing key functions such as mechanical cushioning, thermal insulation, energy reserve, and contouring the body profile [31]. The coordinated interaction of these three layers coordinates the maintenance of the body's internal homeostasis [32]^.^

Wound healing is a coordinated, multi‐phase biological process aimed at the repair of damaged tissue and the subsequent restoration of cutaneous barrier function [33, 34]. Wound healing unfolds through a highly coordinated sequence of four continuous and partially overlapping stages, initiated by hemostasis, followed by inflammation and proliferation, and culminating in tissue remodeling [35]. The initial three stages of wound healing (hemostasis, inflammation, and proliferation) aim to achieve wound closure, restore basic function, and establish the necessary tissue and structural foundation for the subsequent remodeling phase [36]. Consequently, numerous strategies designed to accelerate healing and improve repair quality primarily target these initial three stages (Figure 2B) [37, 38]. However, each phase is defined by distinct cellular and molecular events, which are coordinated to achieve specific physiological goals, with tight linkage and orderly progression between stages collectively ensuring the completion of repair [7]. Therefore, disruption of homeostasis at any stage may derail this highly coordinated sequence, resulting in impaired healing progression and a shift away from regeneration to pathological scarring [39, 40]. A profound understanding of these stage‐specific mechanisms is thus pivotal for revealing the physiological rules of wound healing and explaining the pathological basis of fibrotic diseases.

The hemostatic phase, an immediate response that begins within minutes of injury, is characterized by rapid hemostasis through coagulation and the creation of a provisional matrix, which serves to initiate the entire repair cascade [41, 42]. Upon vascular injury, the triggered platelet activation and aggregation provide an initial physical seal, which subsequently launches the coagulation cascade to convert fibrinogen into fibrin, leading to the eventual formation of a structural fibrin network [43]. In addition to forming a mechanical plug, platelets act as key signaling centers via the release of multiple growth factors (e.g., PDGF, TGF‐β), which initiate healing, modulate inflammation and angiogenesis, and directly modulate fibroblast proliferation and collagen synthesis, thereby collectively driving wound repair [44, 45]. Given these critical roles, the therapeutic potential of platelets has been harnessed ex vivo. Platelet‐rich plasma (PRP), derived from concentrated autologous platelets, is thus regarded as a concentrated reservoir of growth factors. Its mechanism of action involves the exogenous delivery of high‐concentration growth factors to synergistically accelerate tissue repair, modulate inflammation, and suppress pathological scar formation [46, 47].

Spanning from hours to days post‐injury, the inflammatory phase is dedicated to immune defense and the clearance of necrotic tissue, thereby setting the stage for the ensuing repair program [48]. The initial wave of inflammatory cell recruitment involves neutrophils, which are rapidly recruited to the wound and tasked with the clearance of pathogens and necrotic debris [49]. Subsequently, the infiltration of monocytes into the wound is followed by their differentiation into macrophages. Then these cells assume the role of a command center, coordinating the repair process and facilitating the transition to the proliferative phase through performing phagocytic clearance and simultaneously secreting various cytokines and growth factors [39, 50]. The intensity and duration of the inflammatory response exert a dual influence on scar formation [51]. Whereas a well‐controlled inflammatory response facilitates repair, its excessive or prolonged state impedes healing and promotes pathological scarring by upregulating pro‐fibrotic factors (e.g., TGF‐β, TIMP‐1), which disrupt collagen metabolism, induce excessive deposition, and cause aberrant alignment [52, 53]. The inflammatory cytokines IL‐6 and IL‐10 play pivotal roles in modulating the inflammatory response, with IL‐10 promoting healing by attenuating inflammation, whereas the overexpression of IL‐6 is closely associated with pathological scar hyperplasia [54, 55]. Compared to tissues prone to hypertrophic scarring, those that heal with minimal scarring, such as oral mucosa, typically exhibit lower mast cell numbers and reduced activation levels [56]. Excessive inflammation not only delays wound closure but furthermore drives fibrosis, as macrophages in the chronic wound phagocytose the dermal Wnt inhibitor SFRP4, thereby establishing sustained Wnt signaling activity [34, 57].

The proliferative phase signifies the initiation of substantial repair events. Its core processes include the formation of granulation tissue to fill the defect, the re‐establishment of the barrier through re‐epithelialization, and the development of a new vascular network [58, 59]. Fibroblasts act as the key effectors during this stage by migrating to the wound, proliferating, and differentiating into myofibroblasts, which then synthesize and secrete large quantities of ECM components like collagen to build the granulation tissue matrix, thereby providing critical structural support for further repair [60]. However, over‐activation of fibroblasts and the aberrant collagen deposition during this phase represent a primary driver of scar formation [34]. Fibroblast behavior in scar formation becomes uncontrolled, manifesting as excessive activation, differentiation into persistent myofibroblasts, synthesis of disordered ECM, and resistance to clearance [61]. In contrast, fibroblast behavior in scarless healing remains controllable, with precise temporal regulation governing their activation, functional performance, and clearance processes [62]. This ultimately achieves functional regeneration of dermal structure rather than fibrotic repair [63]. Concurrently, activated endothelial cells generate new capillary sprouts to form an extensive microvascular network, thereby supplying the necessary oxygen and nutrients to support the high metabolic demands of the granulation tissue [64]. However, studies indicate that excessive microvascular generation is closely associated with scar formation, while anti‐angiogenic strategies may help reduce scarring [65]. Re‐epithelialization relies critically on keratinocyte migration and proliferation. The restoration of the epidermis, which involves regenerating skin appendages like sebaceous glands, sweat glands, and hair follicles, is closely tied to the mechanisms of scar formation [7]. Beyond their direct role in epithelial regeneration, keratinocytes also modulate the activity of dermal fibroblasts by secreting various signaling molecules such as TGF‐β1, thereby affecting collagen synthesis and deposition [66].

The remodeling phase constitutes the terminal stage of wound repair, serving as the critical period that ultimately dictates scar quality [67]. All prior molecular behaviors and cellular events are transformed at this stage into two ultimate macroscopic outcomes: scarring healing and scarless healing (Figure 2C). The remodeling phase is defined by the wound's shift from reconstruction to maturation, encompassing key processes such as ECM cross‐linking, realignment of collagen fibrils, and regression of the newly formed vessels [68, 69]. The central objective of this phase is to enhance tissue functionality through the optimization and reinforcement of the existing matrix. For instance, the initially deposited type III collagen is progressively replaced by the stronger and more mature type I collagen [70]. Through continuous degradation and resynthesis, collagen fibers undergo active remodeling and align in an orderly manner along the skin's tension lines, thereby optimizing the structure and strength of the healing tissue [71]. However, excessive collagen deposition or impaired degradation leads to its pathological accumulation within the dermis, which constitutes a core pathological feature of fibroproliferative disorders such as hypertrophic scars [72, 73]. Another pivotal event in this phase is the regression of nascent vessels and the establishment of functional microcirculation. However, persistent or incompletely regressed vessels trigger local disturbances in blood supply and metabolism, thereby contributing significantly to the development of pathological scars [65, 74]. Additionally, at the molecular level, sustained TGF‐β signaling during remodeling drives scar pathology, while inhibiting this pathway represents a potential therapeutic strategy for scar reduction [52, 75].

In conclusion, the sequential phases of skin wound healing collectively determine the final morphology of the healed tissue. Abnormal fibrin deposition during the hemostatic phase, dysregulated or prolonged immune responses in the inflammatory phase, aberrant fibroblast activation and imbalanced angiogenesis in the proliferative phase, and disrupted collagen synthesis and degradation in the remodeling phase are all key drivers of pathological scar formation (Figure 2D) [76]. Therefore, an optimal anti‐scarring strategy should shift its focus from counteracting fibrosis to actively promoting regeneration. This entails precise, stage‐specific orchestration of the healing process to restore native skin architecture and physiological function.

## 3 Scarless Regeneration Model

**FIGURE 3:**
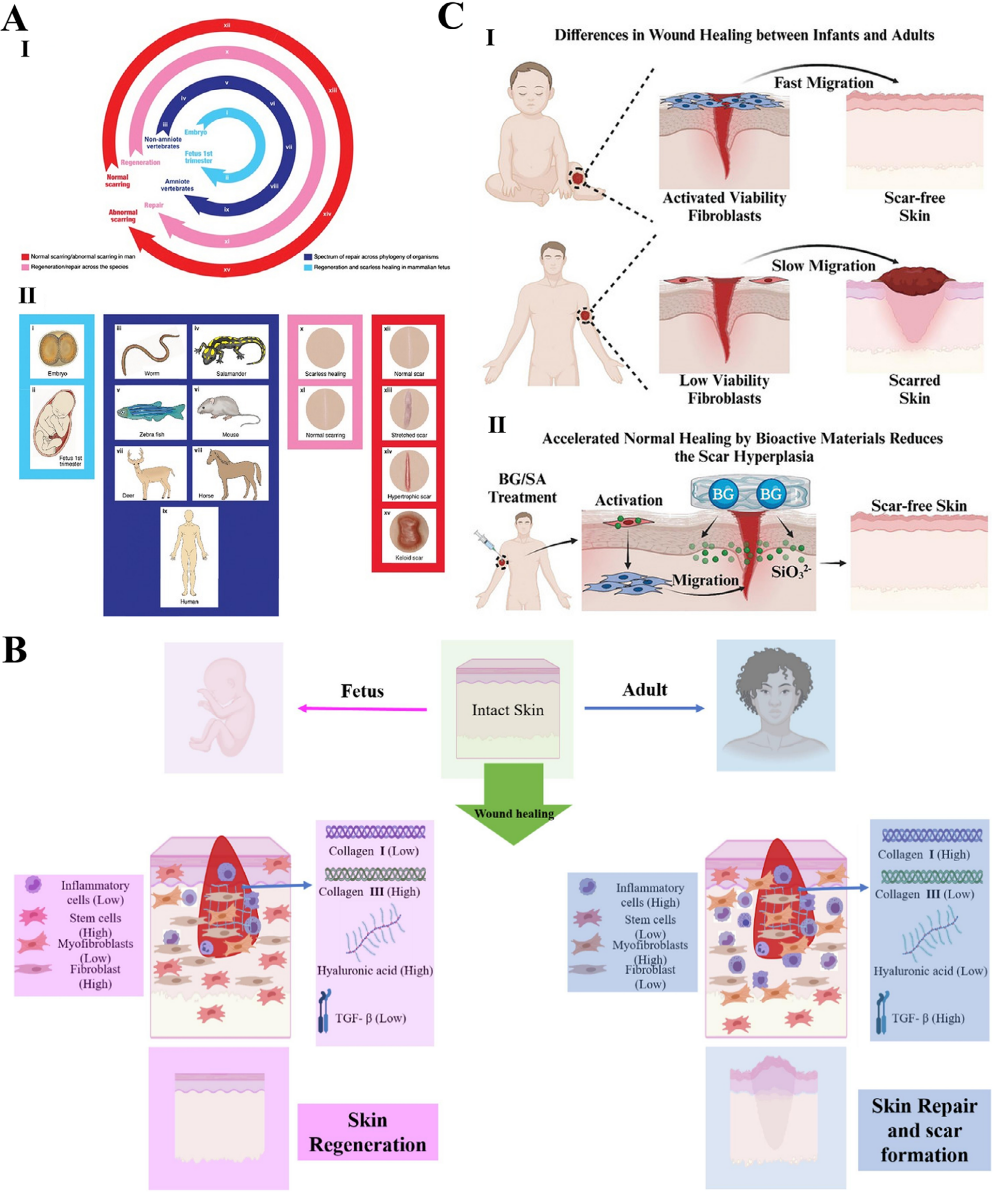
Scarless healing model of fetus. (A) I. The wheel of regeneration: a cross‐species analysis of repair outcomes from scarless Healing to fibrotic scarring, II. The images correspond to the wheels in part (I). Reproduced with permission [79]. Copyright 2014, John Wiley and Sons. (B) Differences in adult and fetus wound healing led to skin repair (scar formation) and regeneration. Reproduced with permission [82]. Copyright 2025, Jonidi Shariatzadeh F, et al., published by Elsevier. (C) A new strategy to inhibit scar formation by accelerating normal healing using silicate bioactive materials. (I) Differences in wound healing between infants and adults, (II) Accelerated normal wound healing by bioactive materials reduces scar hyperplasia. Reproduced with permission [90]. Copyright 2024, Zhang Z, et al., published by John Wiley and Sons.

The response to tissue injury diverges markedly among species, encompassing a broad continuum from complete structural and functional restoration to scar formation characterized by fibrous tissue deposition (Figure 3A) [77, 78, 79]. A representative example is human skin, where repair after significant injury typically culminates in non‐functional scar tissue, resulting in the permanent loss of the original architecture and physiological function [80]. The capacity for scarless healing represents an evolutionarily conserved trait observed in multiple biological systems, notably the early human fetus, *Acomys cahirinus, Ambystoma*, *Xenopus*, and zebrafish [58]. These classic models elicit distinct repair mechanisms that yield regenerated tissue characterized by its native structure and function. A major objective in regenerative medicine is to systematically elucidate the key cellular and molecular differences between species with strong regenerative capacity and those with limited regenerative potential [77, 81]. By leveraging these insights, the core approach involves developing targeted interventions based on tissue‐engineered constructs to precisely coordinate human wound healing, ultimately aiming to replace reparative scarring with functional tissue regeneration.

### 3.1 Scarless Healing in Early Human Fetal Skin

Scarless fetal wound healing represents an ideal model in regenerative medicine, standing in sharp contrast to the fibrotic repair culminating in scar formation observed in adults (Figure 3B) [33]. This process is driven by a highly coordinated regenerative microenvironment that achieves perfect tissue restoration rather than fibrosis through precise regulation of inflammation, cellular activities, and ECM remodeling [82]. Scarless fetal repair is performed within a distinctive intrauterine environment where the wound is continuously bathed in amniotic fluid, which maintains sterility, stable temperature, and a high concentration of growth factors to create an ideal external regenerative microenvironment [83]. Furthermore, fetal wounds exhibit elevated levels of glycosaminoglycans (GAGs), particularly hyaluronic acid (HA) and chondroitin sulfate (CS), compared to adult wounds [84]. This distinct GAG profile helps maintain a highly hydrated, pro‐regenerative microenvironment and supports cell migration and proliferation [85]. The scarless healing in fetal skin is critically dependent on its low‐inflammatory microenvironment, attributable to the underdeveloped immune system [86]. This is characterized by a near absence of neutrophil and macrophage infiltration at the wound site, thereby preventing the tissue damage, oxidative stress, and pro‐fibrotic signaling activation typically triggered by excessive inflammation in adult repair [87]. Consequently, this process eliminates the key driver of pathological scarring at its source. A delicate balance in fetal‐like cellular and molecular phenotypes at the molecular level profoundly influences the regenerative outcome [88]. In fetal wounds, the expression of pro‐fibrotic TGF‐β1/2 is significantly lower than in adults, while TGF‐β3 and FGF, which promote normal proliferation and orderly migration, are relatively highly expressed and play dominant roles [83, 89]. The unique cytokine profile fundamentally alters fetal fibroblast behavior compared to adult cells [89]. These fetal cells proficiently assemble high‐quality ECM characterized by type III collagen‐rich fibrils, while avoiding the excessive proliferation and myofibroblast differentiation seen in adults, thereby preventing disordered collagen accumulation and tissue contraction [83]. By replicating key features of the fetal regenerative environment, including activated dermal fibroblasts, minimal myofibroblast conversion and attenuated inflammation, silicate‐based bioactive materials demonstrate significant efficacy in suppressing scar formation (Figure 3C) [90]. Furthermore, fetal cells themselves possess a strong intrinsic regenerative capacity [91]. Their stem cells and progenitor cells, with their highly undifferentiated and proliferative state, can precisely sense and respond to regenerative signals, thereby efficiently reconstructing complete skin structures with skin appendages [92, 93]. This achieves functional regeneration rather than scar repair. Therefore, designing intelligent biomaterials that imitate fetal ECM characteristics holds promise for precise regulation of immune responses during early healing stages, suppresses excessive inflammation, and reprograms fibroblast phenotypes to reverse their fibrotic tendencies, thereby guiding wounds toward organized regeneration. These regulatory effects are supported by preclinical evidence, though their translation to clinical applications requires further validation [94].

### 3.2 Massive Skin Regeneration in Spiny Mouse (*Acomys cahirinus*)

**FIGURE 4:**
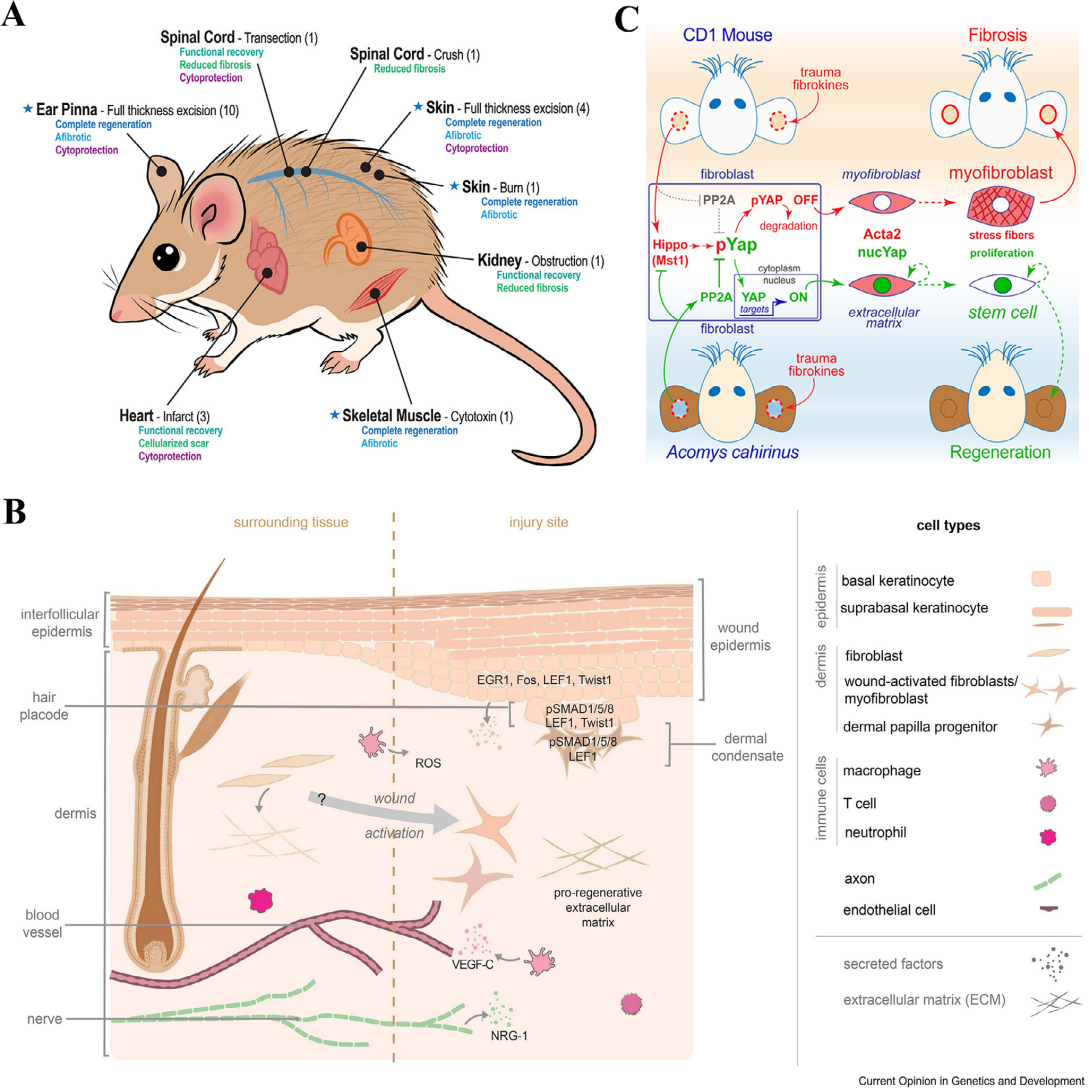
Scarless healing model of spiny mouse(*Acomys cahirinus*). (A) Schematic illustration of the multitissue regeneration of spiny mice. Reproduced with permission [99]. Copyright 2025, The New York Academy of Sciences. (B) Cellular orchestrators of regeneration in spiny mice: key interactions among activated fibroblasts, immune cells (macrophages, T cells), and epidermal cells drive scarless repair, ECM remodeling, and hair follicle neogenesis. Reproduced with permission [100]. Copyright 2024, Tomasso A, et al., published by Elsevier. (C) The mechanistic basis for scarless ear wound healing in spiny mice: adaptations in Hippo‐YAP signaling and myofibroblast fate. Reproduced with permission [104]. Copyright 2021, Elsevier.

Spiny mouse (*Acomys cahirinus*) is the only mammal discovered to date capable of perfect regeneration of large skin areas and multiple tissue types (Figure 4A) [95, 96]. After severe injury, *Acomys cahirinus* skin exhibits accelerated wound closure and progresses beyond mere repair to genuine regeneration, reconstructing functional skin structures comprising hair follicles, dermis, secretory glands, and muscle tissue [97, 98]. The ECM during spiny mouse skin regeneration exhibits extensive hyaluronic acid deposition, and the skin tissue of spiny mouse possesses weak biomechanical properties [98, 99]. This unique soft tissue environment not only provides suitable mechanical and biochemical conditions for cell migration but also underscores the importance of constructing specific regenerative microenvironments. During spiny mouse wound repair, injury signals induce precise regulation of multifaceted interactions among fibroblasts, immune cells, and epidermal cells (Figure 4B) [100]. New hair follicles and glands form within a basket‐like network composed primarily of type III collagen, a specific ECM architecture that represents a hallmark feature of perfect, scarless regeneration [101]. Furthermore, spiny mouse wound healing demonstrates precise immunomodulation, characterized by a rapid transition of macrophage phenotypes and their coordinated interaction with regulatory T cells, which collectively maintain a tightly regulated inflammatory process [102, 103]. Experimental inhibition of YAP in spiny mice prolongs myofibroblast persistence and promotes fibrosis, whereas, forced YAP activation prevents their formation (Figure 4C) [104]. This evidence demonstrates that adaptive evolutionary regulation of YAP activity are pivotal for their scarless regeneration. Thus, the exemplary case of the spiny mouse highlights a promising strategy for mammalian structural regeneration: the coordinated integration of a pro‐regenerative engineering microenvironment, precise control of cell fate, and stage‐specific immune modulation. This strategy is supported by preclinical evidence from spiny mouse models, though its feasibility and efficacy in human wound healing remain to be validated through further translational research.

### 3.3 Tissue Regeneration in Amphibians (*Ambystoma*)

**FIGURE 5:**
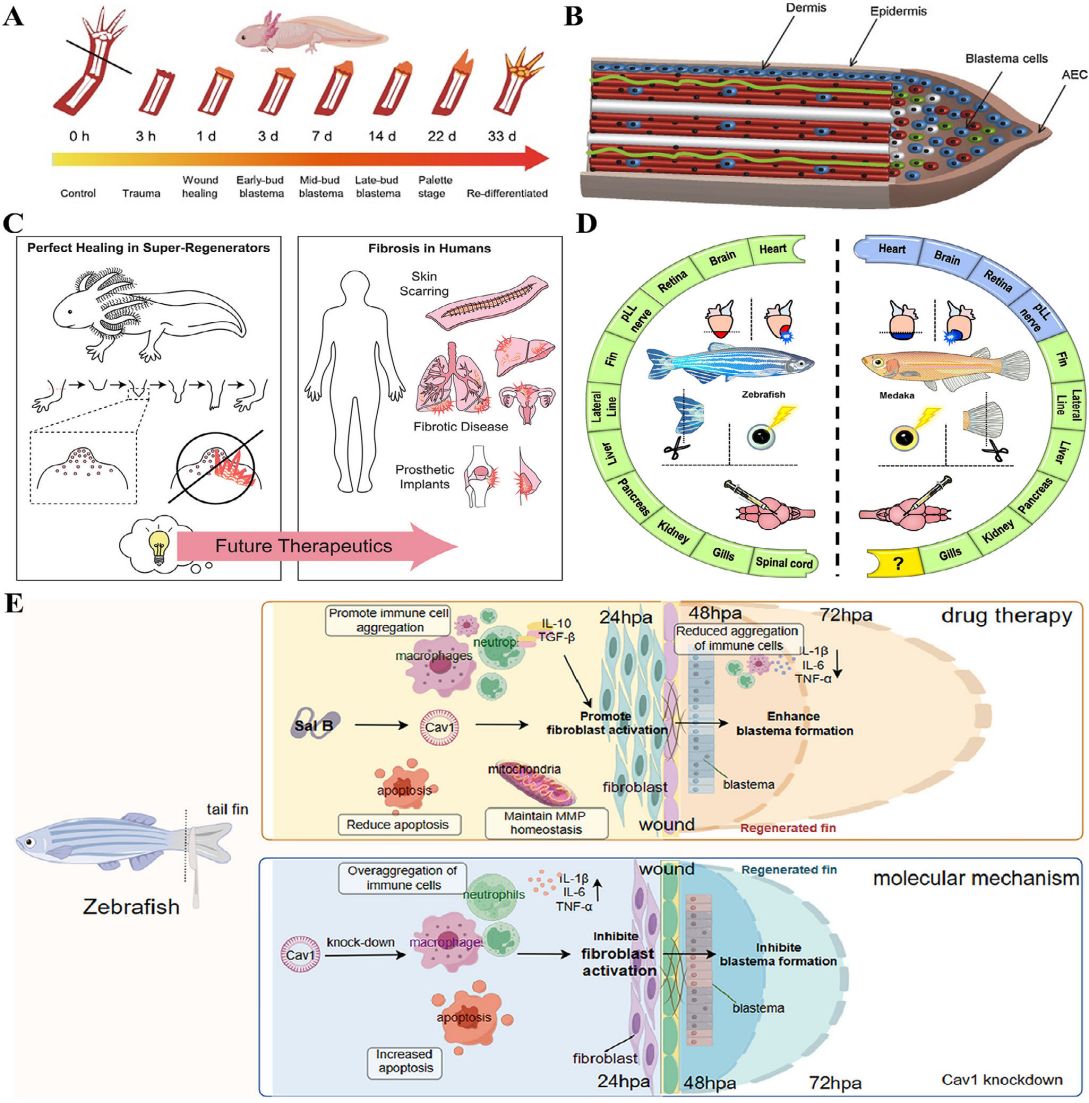
Scarless healing model of *Ambystoma* and zebrafish. (A) The sequential stages of complete limb regeneration in *Ambystoma*, illustrating the extraordinary regenerative capacity of amphibians. Reproduced under terms of the CC‐BY license [106]. Copyright 2020, Li H and Wei X (B) Formation and role of the blastema, the dedifferentiated progenitor cell cluster essential for accurate limb morphogenesis in *Ambystoma*. Reproduced with permission [109]. Copyright 2012, Elsevier. (C) Leveraging the *Ambystoma* limb regeneration blueprint for anti‐fibrotic strategies in humans. Reproduced with permission [115]. Copyright 2021, Durant F and Whited JL, published by John Wiley and Sons. (D) Zebrafish as a vertebrate model for scarless regeneration of multiple organs, including fins, heart, and neural tissue. Reproduced under terms of the CC‐BY license [117]. Copyright 2022, Chowdhury K, Lin S, and Lai S. (E) Salvianolic acid B promotes zebrafish fin regeneration by modulating immune cell migration and Caveolin‐1‐dependent blastema formation, offering a therapeutic model for scarless healing. Reproduced with permission [125]. Copyright 2024, Elsevier.

Amphibians, particularly salamanders, exhibit the most extraordinary regenerative prowess among vertebrates, capable of regenerating complex anatomical structures including complete regrowth of amputated limbs (Figure 5A) [105, 106]. The tissue regeneration of *Ambystoma* is not only characterized by rapid epithelialization, but its core mechanism lies in the successful activation of pluripotent progenitor cells, which serves as the foundation for the complete reconstruction of complex functional tissues [107, 108]. Unlike the limited differentiation potential of mammalian cells, the core advantage of *Ambystoma* lies in cellular fate reprogramming: mature cells within the wounded area undergo dedifferentiation to acquire a progenitor‐like phenotype, ultimately coalescing into a blastema that drives regeneration [109]. This blastema maintains active proliferation while retaining precise positional memory, thereby driving accurate morphogenesis of the regenerating structure (Figure 5B) [110, 111]. Meanwhile, the temporally regulated reactivation of key embryonic signaling pathways, including Wnt, FGF, and BMP, directs blastema cell proliferation, differentiation, and spatial patterning [112]. Wound healing in salamanders not only relies on specific ECM components such as HA but also benefits from high‐concentration release of bioactive peptides (e.g., AMPs) by dermal glands upon injury, and these two factors synergistically enhance the regenerative response [113, 114]. The perfect regeneration mechanism of salamanders not only reveals the importance of integrating multiple factors such as cell fate, signaling timing, and microenvironment, but also offers valuable insights for the development of future strategies to treat and prevent of human fibrotic diseases (Figure 5C) [115].

### 3.4 Complex Tissue Regeneration Model of Zebrafish

The zebrafish serves as a classic model system for studying tissue regeneration in vertebrates, capable of achieving perfect regeneration of multiple organs, including the fin, heart, and neural tissue, among others (Figure 5D) [116, 117]. Zebrafish display an exceptional ability to heal large skin defects, enabling rapid and scarless tissue regeneration without scarring [118]. Unlike zebrafish fin regeneration, which relies on a blastema‐dependent mechanism, skin repair employs a distinct non‐blastemal mechanism initiated by rapid epithelial closure [119, 120]. Rapid epithelialization triggers zebrafish skin regeneration, where keratinocytes execute collective migration through lamellipodial protrusions dependent on functional TGF‐β/integrin signaling [120]. The rapid epithelialization of zebrafish skin also stems from its fin structures serving as a reservoir of migratory epithelial cells. These resident cells possess high migratory capacity, enabling them to rapidly cover wounds in adjacent areas [121]. Zebrafish fins influence skin repair through their inherent regenerative capacity, a process that relies on a highly permissive immune microenvironment [122]. Zebrafish exhibit a rapid and moderate immune response, with their macrophages and T cells displaying a reparative phenotype [123, 124]. Collectively, these factors synergize through the precise temporal regulation of inflammatory signaling, laying the foundation for regeneration. Furthermore, therapeutic modulation that directs immune cell trafficking, attenuates inflammatory infiltration, maintains matrix metalloproteinase homeostasis, and regulates pluripotent progenitor‐mediated blastema formation can effectively promote zebrafish larvae tail fin regeneration (Figure 5E) [125]. Molecularly, the reinstatement of signaling pathways including Wnt/β‐catenin and FGF coordinates blastema cell proliferation and differentiation in a temporally and spatially defined manner during zebrafish regeneration [126, 127]. Notably, its TGF‐β signaling is finely regulated, thereby effectively preventing the onset of a fibrotic response [128]. Thus, the collective modulation of cell reprogramming potential, immune environment, and tightly controlled developmental pathway activation provides a viable strategy for achieving functional tissue regeneration, as evidenced in both zebrafish skin repair and fin regrowth.

### 3.5 Cyclic Regeneration of Deer Antlers

**FIGURE 6:**
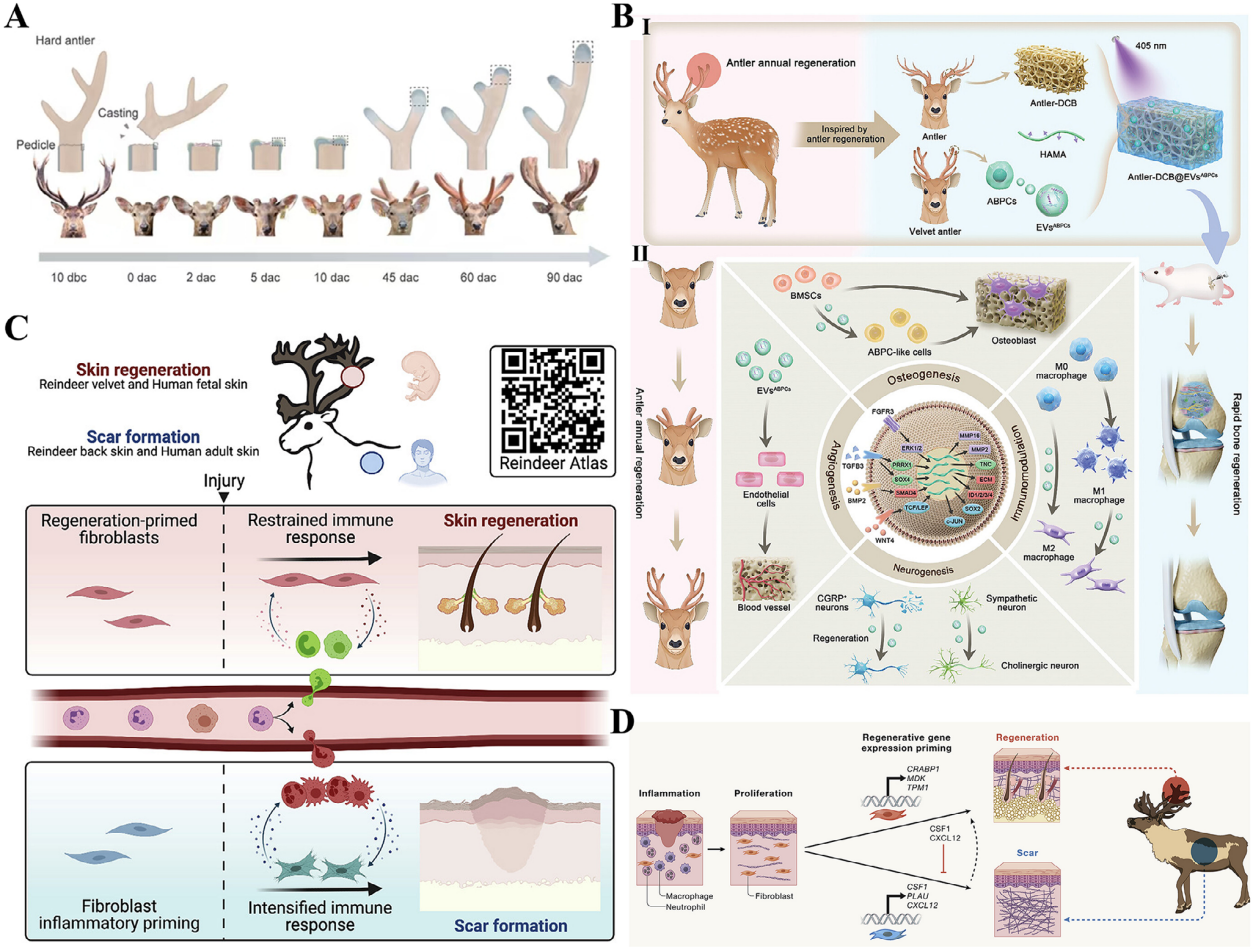
Scarless healing model of deer antlers. (A) The sequential stages of annual, scarless antler regeneration in deer, illustrating a rare mammalian model of organ‐level repair. Reproduced with permission [130]. Copyright 2023, American Association for the Advancement of Science. (B) Deer antler‐inspired bioactive scaffold demonstrates potent osteogenic activity, enabling rapid bone regeneration: I. The antler‐based scaffold mimics the biological characteristics of antlers. II. The antler‐based graft recapitulates key aspects of antlerogenesis, coordinating osteogenesis with angiogenesis, neurogenesis, and immunomodulation to drive rapid bone growth. Reproduced with permission [132]. Copyright 2024, Li S, et al., published by John Wiley and Sons. (C) Fibroblast‐immune cell crosstalk in antler velvet wound healing: A determinant of regenerative versus fibrotic outcomes. Reproduced with permission [133]. Copyright 2022, Sinha S, et al., published by Elsevier. (D) Reindeer fibroblasts exhibit distinct gene activation patterns, driving antler growth and wound healing outcomes in dorsal skin. Reproduced with permission [134]. Copyright 2022, Elsevier.

The cyclical regeneration of deer antlers represents a rare example of organ‐level scarless repair in mammals, which is not mere wound healing but rather a programmed, precisely regulated annual organ regeneration event (Figure 6A) [129]. The core mechanism of antler regeneration lies in the periodic activation of multipotent stem cell clusters in the antler apex. Leveraging their potent proliferative and differentiative potential, these cell clusters rapidly form an embryo‐like mesenchymal structure and differentiate into osteoblasts and other cells, thereby completing the rapid construction of the skeletal framework [130, 131]. Osteo‐inductive grafts bioinspired by antler structural signals demonstrate enhanced recruitment of endogenous BMSCs and their conversion to antlerogenic progenitors [132]. This response coordinates coupled bone deposition with concomitant vascularization, innervation, and immune regulation to enable rapid ossification (Figure 6B). The remarkable plasticity exhibited by cellular behavior suggests the potential for guiding cell fate through targeted interventions to advance tissue regeneration. Additionally, antler velvet tissue damage triggers the formation of task‐specific fibroblast cohorts and remodels the functional state of resident immune cells (Figure 6C) [133]. Following injury, fibroblasts in the dorsal skin of reindeer initiate pro‐inflammatory gene transcription and form scars, a mechanism similar to wound healing in humans and mice, whereas velvet skin achieves regenerative healing through fibroblast‐mediated activation of pro‐regenerative genetic pathways (Figure 6D) [134]. The success of regeneration also depends to a large extent on a highly specialized local microenvironment. The antler tissue contains an abundant, dense vascular network that provides ample nutritional support for cell proliferation [135]. In summary, antler regeneration fundamentally relies on the robust proliferation and differentiation of periosteal stem cells, which is orchestrated by specialized fibroblasts and a pro‐regenerative immune microenvironment. This highlights the critical need for intricate coordination between cellular behavior and the local microenvironment to achieve organ‐level regeneration.

### 3.6 Other Scarless Regeneration Models

Nature presents a series of animal models with distinct scarless healing capacities, including black bears, bottlenose dolphins, genetically modified mice, and annelids [136]. Black bears exhibit slow wound healing with minimal scarring despite prolonged fasting and immobility. This suggests that their unique metabolic state, characterized by low metabolism, weak inflammation, and specific protective protein expression, creates a pro‐regenerative immune microenvironment that may facilitate scarless wound healing [137]. Aquatic mammals, such as the bottlenose dolphin, achieve rapid scarless skin healing in marine environments. This ability is mechanistically linked to the unique immunomodulatory properties of their aquatic environment and a high epidermal turnover rate, indicating that external environmental factors and epidermal homeostasis significantly influence repair [138, 139]. The Aldh1a2 gene‐modified mouse model reveals the genetic regulatory basis of mammalian tissue regeneration [140]. This model exhibits remarkable auricular canal regeneration capacity. The mechanism involves activating endogenous Aldh1a2 genes or exogenous retinoic acid supplementation, which induces fibroblasts to transition to a pluripotent state, thereby reconstructing cartilage and neural tissues within the auricle. This discovery confirms that regulating specific genetic pathways can directly initiate intrinsic regenerative programs within mammals [141, 142]. Lower animal models, such as planarians and annelids, represent the ultimate form of regeneration [143]. The virtually unlimited regenerative capacity of planarians relies on their broadly distributed adult stem cells, precise positional memory system, and precise molecular regulatory mechanisms [144, 145], while certain annelids can reconstitute complete body segments [146]. These diverse scarless models collectively demonstrate that perfect regeneration can be achieved through multiple evolutionary pathways, including physiological adaptation, gene regulation, or complete reconstruction driven by stem cells. A deeper understanding of the underlying mechanisms offers new insights for developing tissue engineering‐based targeted intervention strategies and provides diverse therapeutic targets for clinical scar prevention and treatment.

### 3.7 Comparative Synthesis of Scarless Regeneration Mechanisms

Although the aforementioned scarless regeneration models, encompassing mammals, amphibians, and fish, exhibit distinct evolutionary origins and anatomical structures, comparative analysis reveals striking convergence in their regenerative mechanisms [79]. This convergence not only distinguishes them from the fibrotic wound healing observed in adults but also enables the extraction of conserved regulatory mechanisms, thereby laying the groundwork for translating the scarless phenotype into human therapies (Table 1) [147]. Notably, the clinical translatability of these common mechanisms varies significantly: while some have entered the stage of engineering exploration, others remain primarily as biological observations, facing fundamental challenges to translation (Table 2) [148]. First, in terms of immune regulation, the immune system transitions from passive suppression to active modulation in the scarless model, encompassing immune quiescence and immune plasticity. Early human fetal skin achieves scarless healing through immune quiescence (low inflammation), avoiding pro‐fibrotic signaling cascades [87]. In contrast, species like the spiny mouse (*Acomys cahirinus*), salamanders, and zebrafish employ immune plasticity, actively modulating inflammatory cells and strictly controlling the inflammatory microenvironment to prevent the persistent pro‐fibrotic inflammation typical of adult repair [102, 123]. Notably, mimicking this controlled immune microenvironment represents one of the most clinically promising directions for translation. Engineering approaches, such as the delivery of anti‐inflammatory or regulatory cytokines via biomaterials, have successfully reprogrammed immune phenotypes in animal models, and several smart dressings based on this principle have entered early‐stage clinical exploration [149, 150]. Second, in terms of ECM synthesis, the scarless model exhibits a highly regenerative state. In both fetal wounds and *Acomys cahirinus* wounds, the ECM is primed for regeneration, displaying soft mechanical properties and being rich in hyaluronic acid (HA) [84, 98]. This creates a hydrated, low‐stiffness microenvironment, supporting cell migration and preventing myofibroblast differentiation [85]. This HA‐rich matrix characterizes the blastema‐like structures observed in amphibians and zebrafish, highlighting its evolutionary conservation [151]. In contrast, adult wounds predominantly feature highly rigid, excessively cross‐linked type I collagen, driving persistent fibrosis [73]. Currently, functionalized hydrogels based on HA, collagen, and their derivatives have been extensively applied in preclinical studies [152]. By mimicking the biochemical and mechanical signals of regenerative ECM, they demonstrate clear evidence of promoting organized repair and reducing scar formation in animal models. Several products have already entered clinical application phases, such as DermiSphere hDRT [153]. Third is the divergence in fibroblast fate. Adult healing is characterized by sustained activation and survival of myofibroblasts, with abnormal activity of the key regulatory YAP signaling pathway [154]. In contrast, scarless regeneration in *Acomys cahirinus* is closely associated with sustained YAP signaling (nuclear localization), where myofibroblasts exist only transiently [104]. The minimal number of myofibroblasts in fetal wounds, or the dedifferentiation potential of amphibian embryonic cells, fundamentally hinges on suppressing excessive myofibroblast formation [89]. Currently, targeting signaling nodes like YAP to modulate fibroblast phenotypes has entered an active phase of translational research [155]. For instance, verteporfin, a YAP/TEAD inhibitor, has demonstrated potential for inhibiting fibrosis in laboratory models [156]. However, achieving safe, controllable, and efficient reprogramming of intracellular signaling pathways in human fibroblasts still faces a series of critical challenges, including targeted drug delivery, tissue specificity, long‐term safety, and avoidance of off‐target effects [157]. This field remains in an exploratory phase transitioning toward clinical applications. Fourth is the activation of pluripotent progenitor cells. Species exhibiting scarless healing successfully activate and harness the regenerative potential of endogenous pluripotent progenitor cells to reconstruct intact tissue, demonstrating a high degree of cellular fate plasticity. Salamander cells can dedifferentiate to form a blastema, deer antler wound tissue directly enters a pluripotent state to regain developmental potential, and fetal skin stem cells efficiently reconstruct complete skin architecture, including appendages [109, 130]. In contrast, the stem cell response in adult healing is limited, achieving only epidermal coverage without the reconstruction of complex structures such as dermal papillae and hair follicles [158]. Moreover, dermal fibroblasts tend to rapidly synthesize collagen to fill defects rather than achieving structural regeneration. The clinical translation of this mechanism currently faces fundamental challenges. Despite significant advances in fields like in vitro organoid construction, translational in vivo engineering strategies remain elusive for safely, controllably, and efficiently inducing large‐scale cellular reprogramming or deeply activating endogenous progenitor cells within the complex adult wound microenvironment [158, 159]. Precisely guiding these cells to undergo temporally and spatially ordered morphogenesis signifies the essential leap from repair to regeneration, which still requires breakthroughs in cellular programming and microenvironmental regulation. Finally, the activation and balance of key signaling pathways are crucial. Most notably, this involves subtype switching within the TGF‐β family. Scarless healing consistently involves low expression of pro‐fibrotic TGF‐β1/2 and high expression of anti‐fibrotic TGF‐β3 [83, 89]. The latter promotes type III collagen synthesis by regulating fibroblast activity and prevents excessive myofibroblast activation. In adult scar healing, TGF‐β1 and TGF‐β2 dominate absolutely, while TGF‐β3 expression is low or suppressed. Additionally, in organisms like zebrafish and amphibians, successful reactivation of embryonic or developmental programs (e.g., Wnt, FGF, and BMP) is a common feature, coordinating spatiotemporally precise morphogenesis rather than fibrotic encapsulation [126, 127]. From a translational perspective, reprogramming TGF‐β signaling represents one of the most promising intervention strategies, with related recombinant proteins, neutralizing antibodies, and signal‐modulating biomaterials already entering clinical trials [160]. However, precisely reproducing the complex spatiotemporal expression patterns of pathways like Wnt, FGF, and BMP during regeneration to guide the regeneration of microstructures such as skin appendages and dermal papillae remains a long‐term goal [161]. Existing technologies lack the capacity to achieve such dynamic, fine‐tuned in vivo signal manipulation, constituting a significant technical gap between scar inhibition and achieving complete regeneration.

**TABLE 1:** Comparison of key biological characteristics between scarless regeneration models and adult scar healing.

Key mechanisms	Scarless healing	Adult wound healing (scarring)
Immune profile	Immune modulation (quiescence or plasticity): Restricted activation with rapid reparative switching	Sustained pro‐inflammatory response with persistent neutrophil/macrophage infiltration.
Matrix composition	Pro‐regenerative, HA‐rich, soft matrix with a high Type III to I collagen ratio	Pro‐fibrotic, rigid matrix dominated by densely cross‐linked Type I collagen
Progenitor cell activity	Efficient dedifferentiation and activation of endogenous progenitors	Limited activation of endogenous progenitors and insufficient stem cell response
Myofibroblast dynamics	Low fibroblast differentiation into myofibroblasts, or only transient myofibroblast presence	Persistent myofibroblast activation driving excessive collagen deposition
Signaling network	Dominant TGF‐β3 signaling coupled and controlled YAP activity	Dominant TGF‐β1/2 signaling accompanied by dysregulated, sustained YAP activation
Regeneration results	Perfect structural regeneration with functional restoration	Formation of non‐functional scar tissue, typically accompanied by loss of structure and function

**TABLE 2:** Translational potential evaluation of core mechanisms in scarless regeneration.

Core mechanism	Current engineering progress	Key translational challenges
Immunomodulation [162, 163]	Dressings loaded with immunomodulatory factors and nanomaterials that modulate macrophage phenotypes have been developed	Achieving dynamic, adaptive immune regulation to match the phases of wound healing, mitigating the risk of infection associated with immunosuppression.
ECM biomimetics (Mechanical properties/HA) [164, 165, 166]	Injectable HA hydrogels and mechanically matched scaffolds have been utilized in preclinical studies and some early‐phase clinical trials	Balancing material degradability with tissue ingrowth rates, maintaining long‐term mechanical stability in the dynamic wound microenvironment.
Signaling pathway reprogramming (TGF‐β/YAP) [167, 168, 169]	Local delivery of TGF‐β3, TGF‐β1/2 inhibitors, and YAP pathway modulators (e.g., verteporfin) have been investigated in preclinical studies.	Achieving precise and safe intracellular signal modulation without off‐target effects
Cell fate modulation (Myofibroblasts, Progenitor cells) [170, 171]	Therapies targeting myofibroblast apoptosis or senescence are under exploration, stem cell therapies have been applied in specific clinical scenarios	Safely eliminating pathological cells without compromising normal repair, efficiently and selectively activating and guiding endogenous progenitor cells

In summary, scarless regeneration does not rely on a single pathway but is instead a multi‐layered, programmatic process precisely coordinated by a specific microenvironment. These cross‐species validated common mechanisms, including immune regulation, a soft and HA‐rich ECM, controlled myofibroblast fate, pluripotent progenitor activation, and balanced signaling pathways, collectively form the core blueprint for regeneration. While current clinical and preclinical wound healing strategies offer distinct advantages in accelerating tissue repair and controlling infection, their shared limitation lies in the inability to systematically recapitulate the aforementioned regenerative program, thereby hindering the achievement of true functional scarless healing (Table 3) [172, 173, 174]. This gap highlights the urgency and significant value of developing next‐generation interventions based on scarless healing mechanisms. Consequently, the future trajectory of regenerative medicine is becoming increasingly clear, centered on systematically reconstructing a pro‐regenerative microenvironment through engineered approaches. First, by constructing biomimetic microenvironments, smart materials can be used to simulate the soft, HA‐rich ECM, providing cells with the fundamental physicochemical and mechanical signals that promote regeneration. Second, precise regulation of key cellular fates is achieved through the temporally controlled delivery of signaling molecules (e.g., modulating YAP, delivering TGF‐β3) to reprogram fibroblast phenotypes. This is synergistically combined with immune cell modulation (e.g., inducing macrophage polarization toward a reparative phenotype) to establish an anti‐inflammatory microenvironment and awaken the regenerative potential of endogenous progenitor cells. Further integration of smart materials and advanced manufacturing technologies enables the construction of bioactive 3D scaffolds and responsive delivery systems. These platforms precisely modulate immune and growth signals on demand during dynamic phases of inflammation, proliferation, and remodeling, enabling sequential, multi‐targeted intervention in pathological processes. By drawing inspiration from the convergent evolutionary solutions revealed in these scarless models and integrating cutting‐edge technologies in biomaterials, it is anticipated that next‐generation, highly effective intervention strategies will be developed. This will ultimately drive a fundamental shift in human wound healing, transitioning from scar repair to functional regeneration.

**TABLE 3:** Comparison of advantages and limitations of current wound healing strategies.

Strategy	Key advantages	Limitations
Traditional passive dressings (Gauze, films, non‐adherent dressings) [175, 176]	1. Provides a physical barrier against contamination. 2. Absorbs exudate and maintains a moist wound environment. 3. Low cost, easy to use, and widely accessible	1. Fails to actively promote tissue healing. 2. Requires frequent replacement, carries the risk of secondary trauma during dressing changes. 3. Lacks bioactive signals essential for tissue repair Ineffective in preventing or reducing scar formation
Bioactive materials (GelMA hydrogels, HA hydrogels, collagen‐based scaffolds) [11, 152, 177]	1. Mimics extracellular matrix structure to facilitate cell migration and proliferation. 2. Superior biocompatibility compared to traditional dressings. 3. Modulates the healing microenvironment. Exerts antibacterial and anti‐infective effects	1. Complex material selection and design. 2. Limited clinical applicability. 3. Potential mismatch between degradation rate and tissue ingrowth. 4. Relatively high manufacturing cost. 5. May require specialized storage conditions
Cell and tissue engineering therapies (exosome, acellular dermal matrices, mesenchymal stem cell therapies) [11, 178]	Directly supplements viable cells to enhance regenerative potential. Provides instructive ECM scaffolds for tissue reconstruction. Supports structural regeneration of complex tissues	1. High cost and complex manufacturing processes. 2. Unstable cell survival, integration, and functional performance, potential risks of immune rejection and tumorigenicity. 3. Strict regulatory requirements. 4. Limited clinical accessibility for large‐scale application.
Anti‐scarring agents (Silicone gels, corticosteroids, pressure therapy) [179, 180]	Alleviates hypertrophic scar formation through moisturization, anti‐inflammation, anti‐proliferation, or physical compression. Demonstrated efficacy in improving scar appearance. Easy to apply for long‐term use	Variable efficacy among individuals. Potential side effects with prolonged use. Unable to reverse established pathological fibrosis. Fails to address structural regeneration
Smart dressings (Including 3D/4D bioprinted dressings, theranostic integrated systems, closed‐loop responsive dressings, and wirelessly connected systems) [181, 182, 183]	Real‐time monitoring of wound microenvironment (pH, temperature, etc.) with wireless feedback On‐demand, closed‐loop intervention (antibiotic release, electrical stimulation) 3D/4D bioprinting enables personalized, biomimetic complex structures Integrates diagnosis and treatment, supports remote management, reduces dressing changes Actively modulates wound microenvironment to promote scarless regeneration	High technical complexity and manufacturing cost Balancing biocompatibility, printability, and structural stability is challenging Electronic components face reliability issues (sensor drift, power depletion) Strict regulatory approval pathways and limited clinical validation Requires user training, limited accessibility in resource‐constrained settings 4D bioprinting is still in early‐stage development

## 4 Scarless Wound Healing Tissue Engineering Materials Strategy

### 4.1 Engineering a Biomimetic Microenvironment: ECM‐Based Regulatory Strategies

Traditional scar prevention measures, such as silicone preparations and compression therapy, act via relatively passive mechanisms, primarily improving healing appearance via moisturization, compression, or non‐specific anti‐inflammatory effects, but struggle to achieve structural regeneration [9, 184, 185]. Current research has shifted significantly toward more proactive and fundamental strategies: constructing biomimetic regenerative microenvironments, which aim to emulate the key structure and function of the native ECM and to deliver a precise array of signals that promote regeneration [186, 187]. Characterized by a complex interplay of physical, chemical, and biological factors that maintain a dynamic balance under physiological conditions, the wound microenvironment directly influences the speed and final quality of wound healing [188]. By providing the necessary physical structure and biochemical signals for regeneration, it actively guides tissue toward structural regeneration rather than fibrotic repair [189, 190].

#### 4.1.1 Structural Bionics: Constructing the Physical Framework for Regenerative Spaces

**FIGURE 7:**
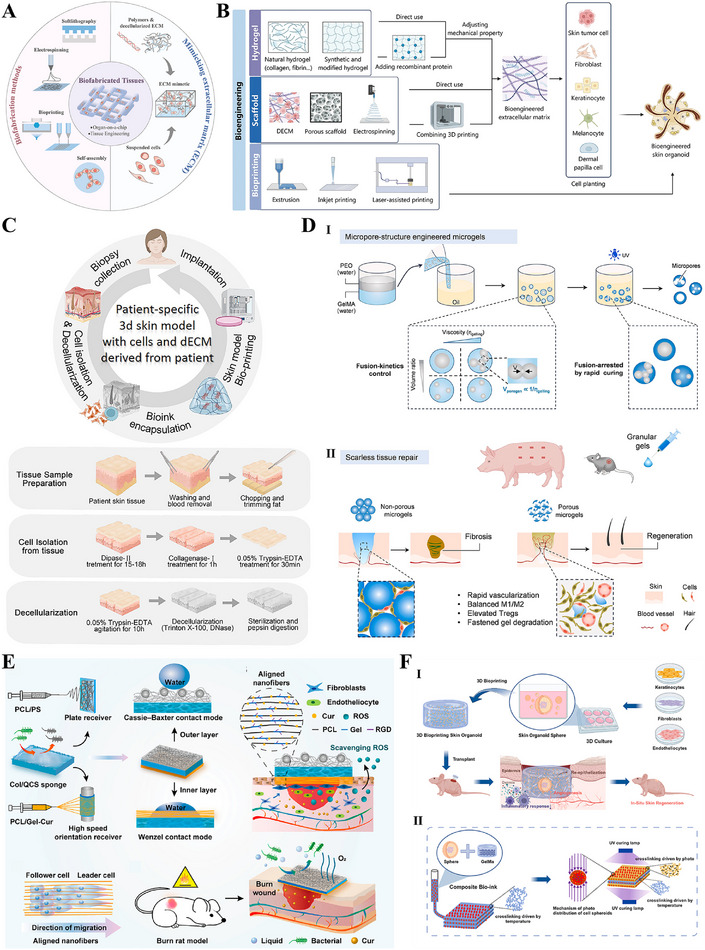
Constructing the physical framework for regenerative spaces. (A) Biofabrication methods for reconstructing ECM mimetics. Reproduced with permission [191]. Copyright 2024, Aazmi A, et al., published by Elsevier. (B) Three bioengineering strategies for regulating the microenvironment and microstructure of skin organoids with precision. Reproduced under terms of the CC‐BY license [194]. Copyright 2023, Hong Z, et al. (C) Bioengineered skin graft integrating patient‐derived decellularized ECM with autologous cells to construct a personalized, biomimetic regenerative microenvironment. Reproduced with permission [195]. Copyright 2025, Kang R, et al., published by John Wiley and Sons. (D) Micropore‐structured hydrogel fabricated via advanced engineering promotes scarless regeneration by enhancing cell migration, modulating inflammation, and inducing vascularization. Reproduced with permission [199]. Copyright 2025, Elsevier. (E) An electrospun biomimetic asymmetric dressing with aligned nanofibers promotes healing in severe burn wounds by directing cell migration and resisting infection. Reproduced with permission [203]. Copyright 2022, American Chemical Society. (F) 3D bioprinting enables the fabrication of cell‐laden, biomimetic skin organoids that accelerate in situ regeneration and healing of full‐thickness skin defects. Reproduced under terms of the CC‐BY license [206]. Copyright 2024, Zhang T, et al., published by Elsevier.

Structural bionics focuses on constructing physical scaffolds with defined topology, controllable mechanical properties, and multi‐scale porosity. These scaffolds provide a supportive and instructive 3D microenvironment for cell growth, tissue remodeling, and regeneration (Figure 7A) [191]. By simulating the physical characteristics of the ECM, bionic scaffolds reproduce its multidimensional characteristics, thereby enabling the loading of various skin‐associated cells, the formation of skin organoids, and ultimately the promotion of complete skin regeneration (Figure 7B) [192, 193, 194]. Representative systems include decellularized ECM scaffolds, hydrogel networks, fibrous membranes, and 3D‐bioprinted structures. Decellularized ECM scaffolds are derived from natural tissues and retain key ECM components (such as collagen, fibronectin, laminin, and various growth factors), providing cells with a highly biomimetic habitat [195, 196]. The integration of patient‐derived decellularized ECM (pddECM) and a keratin‐alginate (KA) bioink has enabled the construction of a biomimetic skin microenvironment [195]. This microenvironment not only replicates the composition and structure of native skin but also coordinates ECM remodeling, angiogenesis, and anti‐fibrotic activity, thereby promoting functional tissue regeneration (Figure 7C). Hydrogel scaffolds with microporous structures can precisely construct ECM‐like 3D spaces and serve as multifunctional cell or drug carriers, demonstrating significant application potential in tissue engineering and regenerative medicine [197, 198]. Researchers have discovered that the microporous structure of hydrogels not only promotes active migration and infiltration of host cells but also suppresses excessive inflammatory responses through physicochemical synergistic mechanisms, inducing mature vascular network formation and achieving scarless regeneration (Figure 7D) [199, 200]. Advanced manufacturing techniques, particularly electrospinning and 3D printing, provide superior conditions for the precise fabrication and functional design of skin‐mimetic scaffolds [82, 201]. Electrospinning technology enables the efficient fabrication of fibrous scaffolds that replicate the nanofibrous topology of the native ECM. By precisely controlling fiber diameter, alignment, and porosity, such scaffolds can accurately simulate the physical microenvironment of natural tissues, thereby providing crucial physical guidance for cell adhesion, migration, and tissue regeneration [184, 202, 203]. A biomimetic asymmetric dressing combining an oriented hydrophilic nanofiber inner layer for directed cell migration and a micro‐nanostructured superhydrophobic outer layer for bacterial anti‐adhesion, both prepared via electrospinning, creates a healing‐promoting microenvironment [203]. In severe burn models, it demonstrates synergistic enhancement of epithelial regeneration, angiogenesis, and infection management (Figure 7E). 3D printing, and specifically bioprinting, offers unprecedented geometric freedom and spatial control in manufacturing biomimetic scaffolds, allowing for the precise, layer‐by‐layer assembly of structures with intricate internal architectures [204, 205]. Researchers have innovatively utilized extrusion‐based bioprinting integrated with dual‐light source cross‐linking technology to construct cell‐laden skin organoids [206]. This biomimetic microenvironment can be customized to match wound morphology and has been demonstrated in mouse models to effectively accelerate in situ regeneration and healing of full‐thickness skin defects (Figure 7F). In addition to constructing wound microenvironments, researchers have also utilized single‐cell coating and 3D bioprinting technologies to create highly biomimetic skin 3D microenvironments, confirming that this environment can regulate the quiescence/activation of hair follicle stem cells, restore the inductive capacity of dermal papilla cells, and consequently promote hair follicle neogenesis and skin hair regeneration [207, 208, 209].

In summary, structural bionics have evolved from merely providing physical support to serving as an intelligent strategy for constructing microenvironments that actively guide cellular behavior and tissue regeneration. Supported by accumulating preclinical evidence, technologies such as hydrogels, decellularized matrices, electrospinning, and 3D bioprinting enable researchers to precisely replicate the physical topography and mechanical properties of the natural extracellular matrix. This precise control has been demonstrated to effectively modulate key regenerative processes, including cell infiltration, vascular network formation, and inflammatory responses, thereby establishing a robust preclinical foundation for achieving complete functional skin regeneration, including its appendages. However, translating these structural biomimetic strategies from preclinical models to clinical applications, particularly achieving consistent appendage regeneration and long‐term functional stability, remains a critical challenge requiring further validation. Future research should focus on optimizing material design, enhancing biocompatibility, and elucidating the underlying mechanisms of cell‐material interactions, with the long‐term goal of realizing the clinical application potential of structural biomimicry in functional skin regeneration.

#### 4.1.2 Compositional Bionics: Constructing the Biochemical Foundation for Regenerative Signals

**FIGURE 8:**
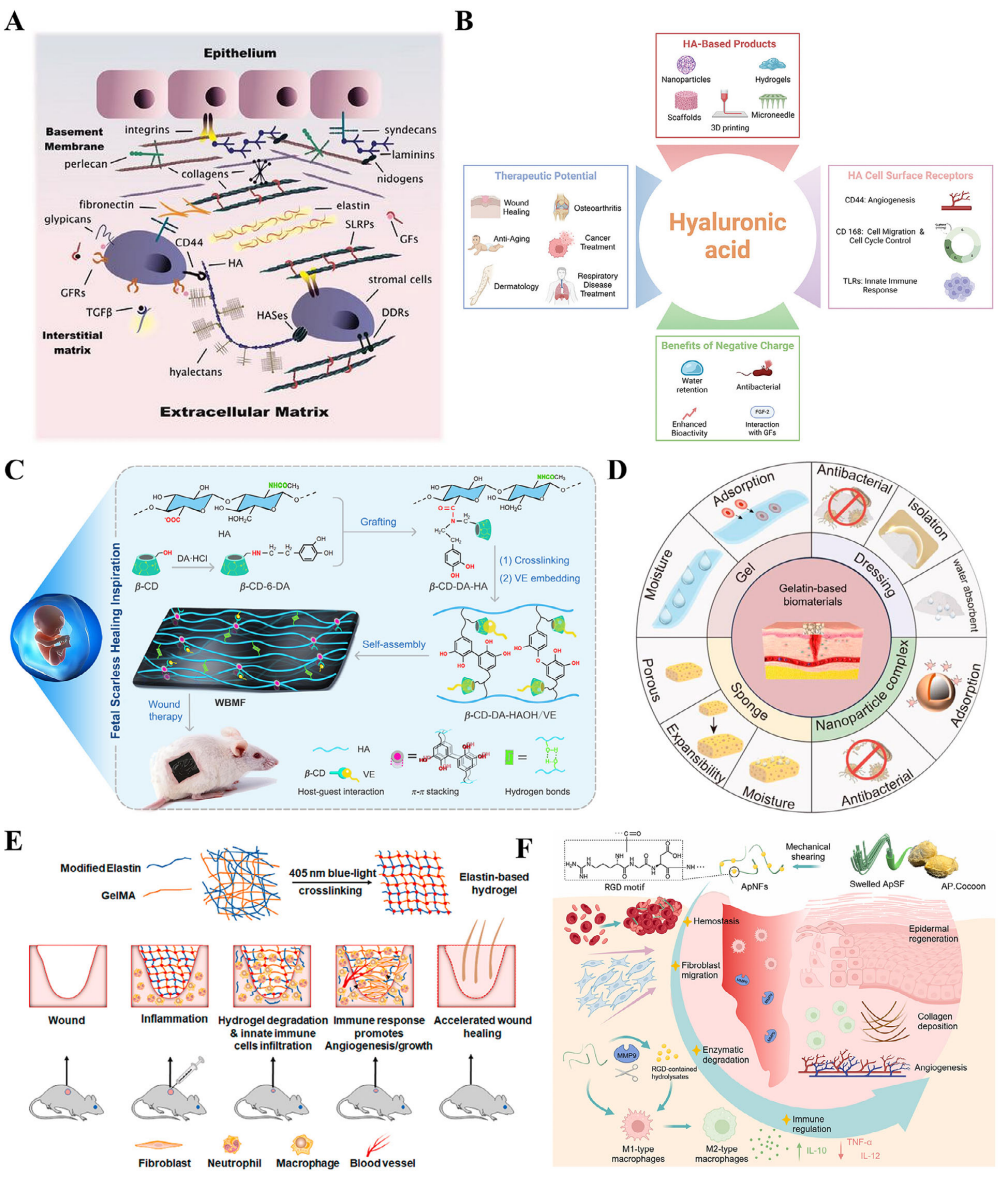
Constructing the Biochemical Foundation for Regenerative Signals. (A) Key biochemical components of the native extracellular matrix (ECM), including collagen, hyaluronic acid, elastin, and fibrin, that provide the foundation for compositional bionics. Reproduced with permission [210]. Copyright 2021, Federation of European Biochemical Societies. (B) Schematic illustration of the biomedical potential of HA. Reproduced with permission. [213]Copyright 2024, John Wiley and Sons. (C) An HA‐based wearable biomimetic membrane replicates key features of fetal ECM to achieve scarless dermal repair by providing a sterile, moist, and pro‐regenerative microenvironment. Reproduced with permission [187]. Copyright 2020, American Chemical Society. (D) Gelatin, derived from collagen, is a versatile biomaterial favored for wound healing applications due to its biocompatibility, processability, and capacity for biofunctionalization. Reproduced under terms of the CC‐BY license [215]. Copyright 2024, Cao H, Wang J, Hao Z, Zhao D. (E) Elastin‐gelatin hydrogels mimic the dermal microenvironment and, upon degradation, release peptides that coordinate immune responses and stimulate angiogenesis to enhance dermal regeneration. Reproduced under terms of the CC‐BY license [216]. Copyright 2022, Tian D, et al., published by Elsevier. (F) Injectable silk nanofibrils with inherent RGD motifs promote diabetic wound healing by accelerating hemostasis, regulating immunity (M1 to M2), and improving re‐epithelization without exogenous drugs. Reproduced with permission [220]. Copyright 2025, Elsevier.

The ECM serves not merely as a physical scaffold for cellular support, but as a complex network of bioactive molecules whose biochemical properties dynamically regulate key cellular behaviors including adhesion, proliferation, differentiation, and migration(Figure 8A) [210]. In terms of compositional bionics, the use of natural biomimetic materials is one of the key strategies for constructing regenerative microenvironments [187, 189]. Components widely present in the ECM, such as collagen, HA, elastin and fibrin, have been extensively applied due to their superior biocompatibility, degradability, and ability to mimic the biological functions of natural extracellular matrices [194, 211, 212]. As a representative glycosaminoglycan of the ECM, HA has attracted considerable research interest. This is primarily attributed to the discovery of its key role in scarless wound healing in fetuses, and second, to its negatively charged molecular structure and ability to bind to various cell surface receptors, which underlies its promising therapeutic potential across diverse scenarios (Figure 8B) [213]. Based on these properties, HA is frequently incorporated as a key functional component in the biomimetic design of tissue engineering materials, aiming to replicate and reconstruct this pro‐regenerative microenvironment in multiple tissue repair fields [214]. Fetal ECM‐inspired biomimetic hydrogels based on HA and chondroitin sulfate reproduce the glycosaminoglycan‐rich nature of native tissue, synergistically enhancing angiogenesis and hair follicle regeneration in experimental models to enable efficient skin wound healing [189]. Beyond replicating the glycosaminoglycan‐enriched properties of the ECM, researchers have developed a wearable biomimetic membrane based on HA and other components by simulating the sterile, moist environment and key biological functions of fetal ECM, effectively achieving scarless repair of the dermis (Figure 8C) [187]. Although structural ECM proteins such as collagen, fibrin, and elastin have all been utilized in constructing biomimetic scaffolds, gelatin has emerged as the most widely used material in tissue engineering, owing to its superior biocompatibility, excellent processability, and superior capacity for incorporating bioactive molecules (Figure 8D) [215]. Gelatin‐based microneedles (GMNs) have emerged as a versatile platform for scar management [10]. Their array‐based microstructure allows minimally invasive delivery of a broad spectrum of therapeutics (small molecules, growth factors, siRNA, exosomes, etc.) to be precisely delivered to dermal sites, effectively preventing and inhibiting scarring during wound healing [20]. Furthermore, researchers have created tunable bioactive elastin‐gelatin hydrogels that mimic the dermal microenvironment [216]. Degradable by neutrophil elastase, these hydrogels release elastin‐derived peptides which coordinate immune responses and boost angiogenesis, leading to enhanced dermal regeneration and collagen deposition in mice (Figure 8E). Adhesive proteins (e.g., fibronectin, laminin) and their derived bioactive peptides (e.g., the RGD peptide) within the ECM act as key signaling molecules that regulate the wound healing process [217, 218]. By mediating essential cellular behaviors, such as adhesion, migration, proliferation, and differentiation, and modulating the activity of growth factors, they work in concert to ensure efficient and orderly tissue regeneration, ultimately influencing the wound healing outcome [219]. Researchers have developed injectable and degradable nanofibrils from *Antheraea pernyi* silk, which leverage their inherent RGD motifs to accelerate diabetic wound healing by promoting coagulation, fibroblast migration, and macrophage polarization from M1 to M2 phenotypes, thereby modulating chronic inflammation and reducing MMP‐mediated tissue degradation without the need for exogenous drugs(Figure 8F) [220].

In summary, the core premise of conformational biomimicry lies in the growing body of preclinical evidence supporting the notion that natural ECM components, such as HA and gelatin, can serve as biological signals to construct bio‐oriented microenvironments. Consequently, an emerging consensus holds that the future of regenerative medicine lies in the strategic synergy between structural and conformational biomimicry. While this integrated paradigm is theoretically crucial for fully mimicking the complexity of the native ECM, and preclinical studies have demonstrated its potential in regulating key physiological processes (e.g., immunomodulation, directed cell migration), achieving consistent scar‐free healing and functional tissue regeneration remains a goal requiring further validation. Notably, translating these synergistic strategies from preclinical models to clinical applications faces unresolved challenges, such as optimizing the precise spatiotemporal coordination of structural and configurational cues. Future research should focus on elucidating the underlying mechanisms of crosstalk between material structure and composition, with the long‐term goal of realizing the clinical potential of biomimetic strategies in functional tissue regeneration.

### 4.2 Regulating Key Cellular Fates: Intervention Strategies Based on Signaling Molecules and Cellular Interactions

Despite their focus on delivering growth factors, scaffolds, and infection control, conventional wound therapies frequently fail to achieve a fundamental reversal of pathological cellular states within the wound [221, 222]. Recent studies have progressively moved toward attaining functional regeneration by modulating cell fate [90, 223]. Induced cell reprogramming is now a coordinated strategy that aims to control cell plasticity by integrating material design, release kinetics, and microenvironment modulation. This allows it to mimic the natural plasticity seen in regeneration models such as spiny mice and antlers [188, 224, 225, 226].

#### 4.2.1 Exogenous Intervention: Introduction of External Cells or Cellular Components

**FIGURE 9:**
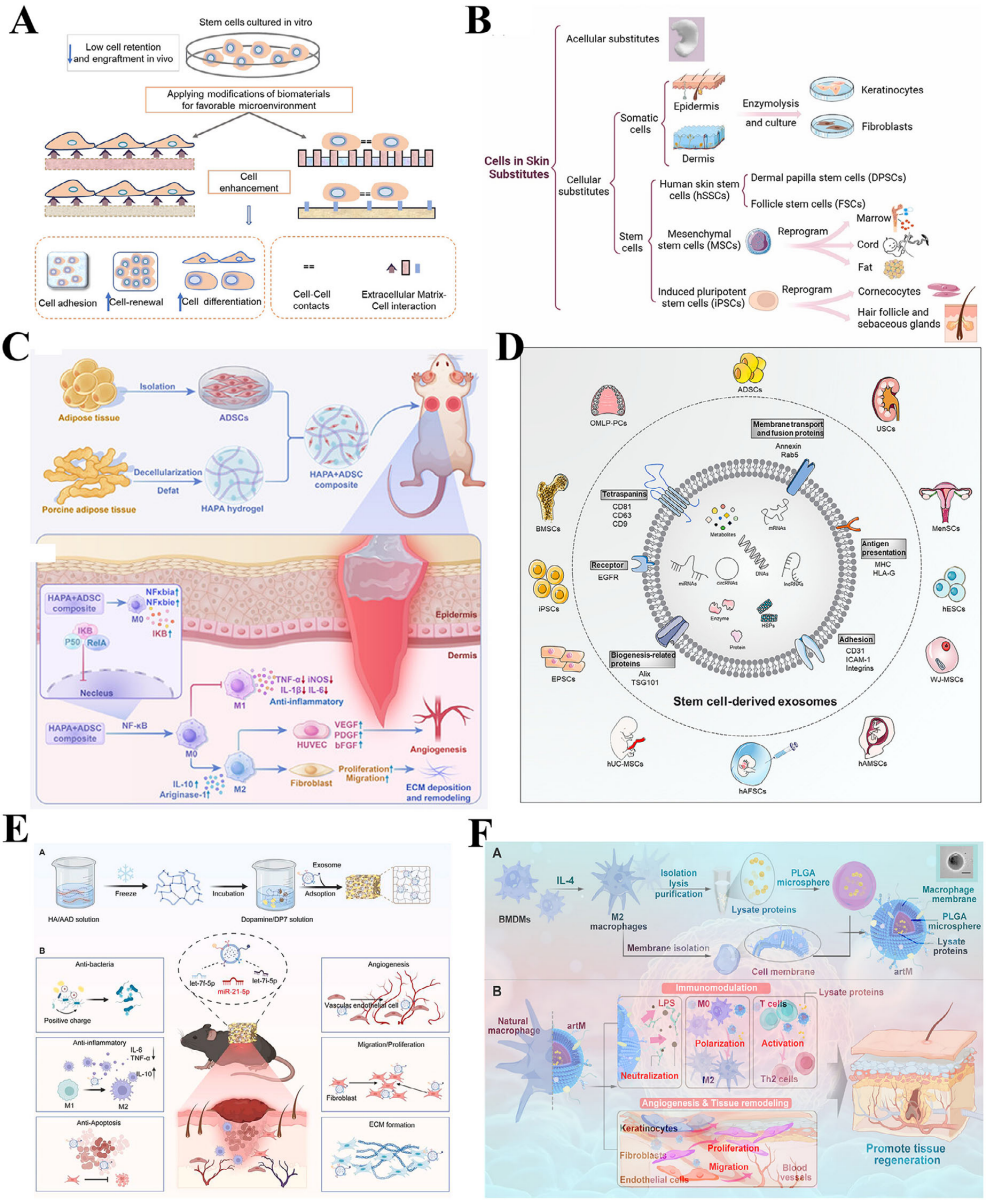
Intervention involving the introduction of external cells or cellular components for skin regeneration. (A) Exogenous cell and component introduction strategies to enhance artificial scaffolds by providing building blocks or instructive cues for tissue repair. Reproduced under terms of the CC‐BY license Reproduced with permission [229]. Copyright 2021, Zhao X, et al. (B) Cell types referenced in the application of skin wound dressings. Reproduced with permission [230]. Copyright 2024, Zhang M, et al., published by Elsevier. (C) A composite hydrogel of extracellular adipose matrix and adipose‐derived stem cells for enhanced wound repair via macrophage polarization. Reproduced with permission [233]. Copyright 2025, American Chemical Society. (D) The compositions, biomarkers, and sources of stem cell‐derived exosomes, which modulate the wound microenvironment through bioactive cargo delivery. Reproduced with permission [178]. Copyright 2023, Zhou C, et al. (E) Exosome hydrogel enables multifunctional scarless healing via miR‐21‐5p by coordinately modulating interactions among multiple cell types and inhibiting myofibroblast differentiation. Reproduced with permission [237]. Copyright 2024, Elsevier. (F) Artificial macrophages, assembled from PLGA microspheres encapsulated by macrophage membranes, recapitulate endogenous macrophage functions to promote skin regeneration via immunomodulation and angiogenesis. Reproduced with permission [240]. Copyright 2025, Su Q, et al., published by John Wiley and Sons.

When the repair capacity at the injury site is insufficient or dysregulated, the exogenous intervention strategy, which involves providing external assistance, demonstrates significant application potential [227]. As a core exogenous intervention strategy, introducing exogenous cells and their components aims to enhance biomaterials by serving as building blocks or delivering guidance cues to create a favorable microenvironment, thereby actively guiding and accelerating tissue repair (Figure 9A) [228, 229]. Multiple types of exogenous cells, including mesenchymal stem cells, fibroblasts, and human umbilical vein endothelial cells, have been incorporated into biomaterials, thus forming composite dressings or scaffolds that provide both structural support and bioactivity (Figure 9B) [230]. Exogenous cells can directly differentiate to replenish required cell types, and additionally exert immunomodulatory effects through paracrine activity to reduce inflammation and promote cell migration and proliferation, thereby synergistically optimizing healing outcomes (Figure 9C) [231, 232, 233]. Beyond the direct effects of introduced cells, other researchers have demonstrated that the combined application of stem cell‐laden hydrogel microspheres and sustained acupuncture therapy can effectively modulate the interactions among fibroblasts, macrophages, endothelial cells, and keratinocytes, thereby guiding the skin toward regenerative healing rather than scar formation [234]. Furthermore, in addition to using whole cell transplants, significant efforts are directed toward harnessing cell‐derived components, like exosomes, cell membranes, cellular nucleic acids and bioactive factors, which serve as cell core functional entities that directly deliver complex biological instructions to initiate endogenous regeneration [36, 60]. Compared to direct stem cell transplantation, stem cell‐derived exosomes exhibit lower immunogenicity and superior stability [235]. Their inherent capacity as natural biological messengers enables them to precisely modulate the wound microenvironment through the delivery of bioactive cargo, including diverse proteins and nucleic acids (Figure 9D) [178]. Single‐cell sequencing revealed that human adipose‐derived mesenchymal stem cell (ADSCs)‐derived exosomes coordinate scarless repair by targeting the 14‐3‐3 zeta‐YAP‐Hippo pathway to suppress fibrosis, while simultaneously reprogramming keratinocyte plasticity and fibroblast crosstalk to mitigate scarring [236]. Beyond exosomes derived from adipose stem cells, researchers demonstrated that exosomes derived from mesenchymal stem cell also can coordinately modulate the interactions among fibroblasts, endothelial cells, and macrophages [237]. This effectively inhibits myofibroblast differentiation and fibrosis, thereby enabling tissue regeneration with reduced scarring in burn model (Figure 9E). Moreover, by coating synthetic scaffolds with natural cell membranes (e.g., from stem cells, immune cells, or platelets), materials can acquire the identity and key functions of the parent cells, enabling advanced properties like immune evasion, active targeting, and signal modulation [238]. Studies have revealed that a myofibroblast membrane‐coated nano‐platform can direct diverse cellular fates [239]. It not only clears bacteria through membrane camouflage and reprograms macrophages to an M2 phenotype for inflammatory resolution, but also releases membrane components to epigenetically inhibit fibroblast‐to‐myofibroblast transformation, ultimately accelerating skin wound healing. Artificial macrophages encapsulating PLGA microsphere cores within macrophage membranes recapitulate the fundamental inflammatory regulatory and regenerative properties of endogenous macrophages [240]. They promote tissue regeneration through immunomodulation, angiogenesis, scar minimization, and skin appendage renewal (Figure 9F). Additionally, the use of delivery platforms loaded with specific miRNAs to exert local immunomodulatory and anti‐fibrotic effects, though still in the exploratory phase, has shown potential value [241]. In brief, exogenous cells and their components can act as building blocks to replenish tissues or serve as guidance cues to actively create a regenerative microenvironment through paracrine signaling, immunomodulation, and regulation of key pathways, thereby guiding scarless repair. Future research will continue to focus on elucidating the precise mechanisms of action of cellular components and developing intelligent delivery systems and biomimetic materials to achieve more precise and efficient exogenous interventions.

#### 4.2.2 Intrinsic Reprogramming: Redirecting the Fate of Resident Wound Cells

**FIGURE 10:**
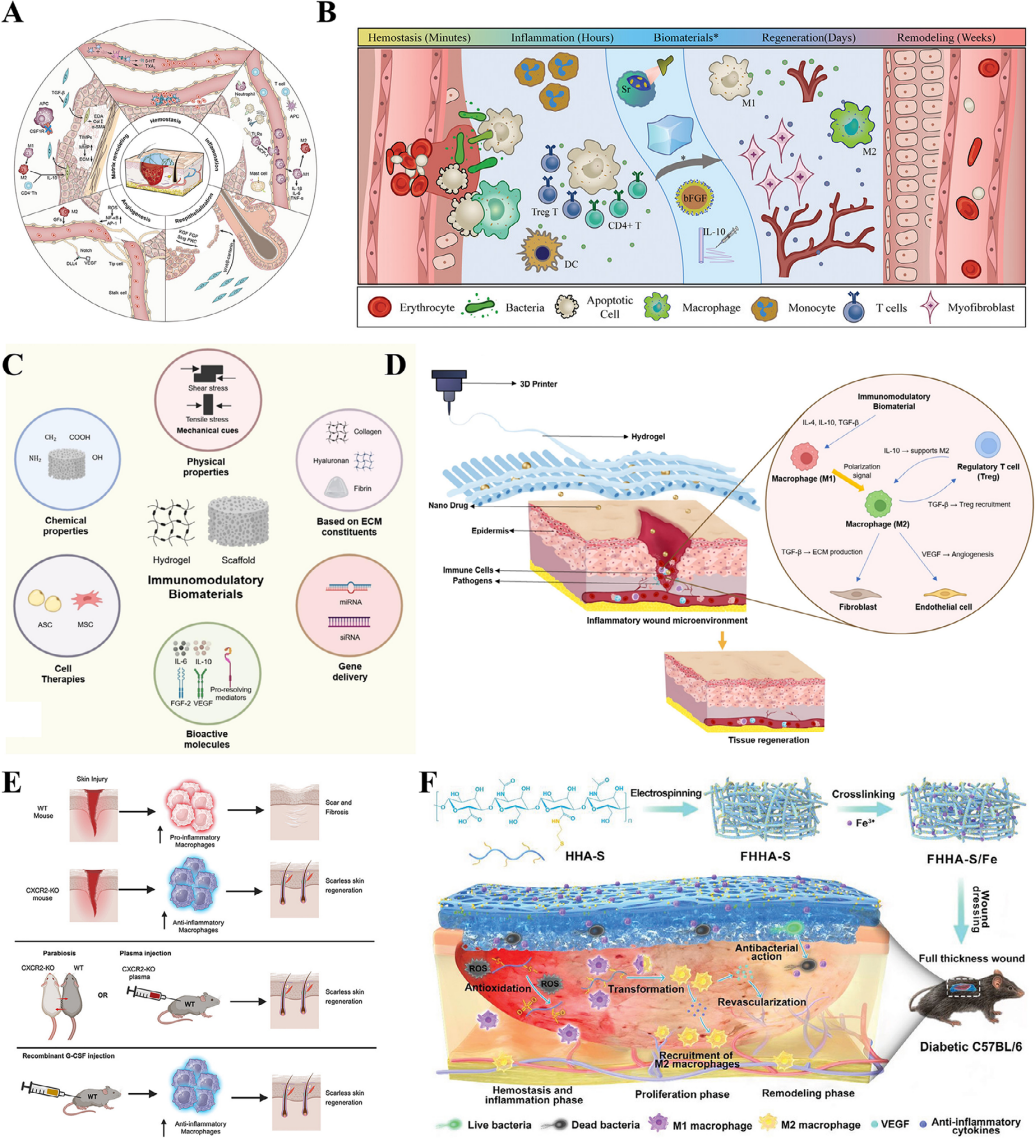
Regulating the fate of inflammatory cells to guide skin regeneration. (A) Bioactive materials reprogram the fate of resident wound cells (e.g., fibroblasts) to break the fibrosis cycle and promote functional tissue regeneration. Reproduced under terms of the CC‐BY license [242]. Copyright 2022, Li R, et al., published by John Wiley and Sons. (B) Schematic illustration depicting the normal wound healing process, highlighting the pivotal role of regulated macrophage responses in steering outcomes toward scarless regeneration. Reproduced with permission [244]. Copyright 2021, John Wiley and Sons. (C) Schematic illustration of immunomodulatory biomaterials and their mechanisms of action to actively regulate the inflammatory response for wound healing. Reproduced with permission [245]. Copyright 2025, Elsevier. (D) Schematic illustration of immunomodulatory pathways in advanced biomaterials, which transform the wound microenvironment to achieve scarless tissue regeneration. Reproduced with permission [245]. Copyright 2025, Elsevier. (E) Granulocyte colony stimulating factor facilitates scarless tissue regeneration by polarizing macrophages toward an anti‐inflammatory phenotype and recruiting them to the injury site. Reproduced with permission [246]. Copyright 2024, Elsevier. (F) Absorbable HA‐based hydrogel accelerates wound healing by reprogramming macrophage polarization from M1 to M2 phenotype. Reproduced with permission [248]. Copyright 2020, John Wiley and Sons.

Driven by recent advances in regenerative medicine, the revolutionary model of intrinsic cell reprogramming has emerged. Instead of introducing exogenous stem cells, this approach directly reprograms the fate of resident cells (e.g., fibroblasts, immune cells) within the wound niche (Figure 10A) [242, 243]. By directly engineering these core players in the local microenvironment, it can more precisely and sustainably break the vicious cycle of fibrosis and promote the genuine regeneration of functional tissue [7].

A notable feature of various scarless wound healing models is a mild inflammatory response, in contrast to scarring healing, which often progresses to scar formation due to dysregulated inflammation [87]. The inflammatory response spans the entire wound healing cascade, and regulating its magnitude, timing, and cellular constituency is pivotal for steering the outcome toward scarless regeneration (Figure 10B) [244]. Currently, a variety of immunomodulatory materials have been designed to actively regulate the inflammatory response during wound healing through multiple mechanisms, including physical properties (e.g., stiffness and topography), bioactive components (e.g., ECM components, signaling molecules, and cell therapies), and chemical properties (e.g., surface charge and functional groups) (Figure 10C) [244, 245]. By leveraging advanced technologies such as 3D printing, immunomodulatory biomaterials are engineered that release specific signals (e.g., interleuki‐n10 (IL‐10), TGF‐β) [245]. This transforms the wound microenvironment from pro‐inflammatory to pro‐regenerative, and by directing the functions of immune cells like macrophages, ultimately achieve scarless tissue regeneration (Figure 10D). For instance, the cytokine IL‐10 can promote the M2 polarization of macrophage, which alleviates the inflammation primarily driven by the M1 macrophage subset at the injury site and facilitates effective wound healing [59]. Granulocyte Colony‐Stimulating Factor derived from the blood of mice lacking the C‐X‐C motif chemokine receptor 2 (CXCR2) can directly polarize macrophages toward an anti‐inflammatory phenotype and mediate their robust recruitment to the injury site, thereby promoting scarless tissue regeneration (Figure 10E) [246]. Beyond the direct administration of exogenous cytokines, leveraging the intrinsic properties of biomaterials to modulate the immune microenvironment serves as another promising strategy for regulating inflammatory cells [247]. Even without the incorporation of additional active factors, various scaffold dressings can effectively promote the phenotypic conversion of aggregated M1 macrophages to the M2 phenotype (Figure 10F) [247, 248]. This shift coordinates an accelerated transition of the wound healing process from the inflammatory phase to the proliferative and remodeling stages, demonstrating that the intrinsic properties of the materials themselves are an effective means of intervening in inflammatory cell fate and improving the regenerative microenvironment.

**FIGURE 11:**
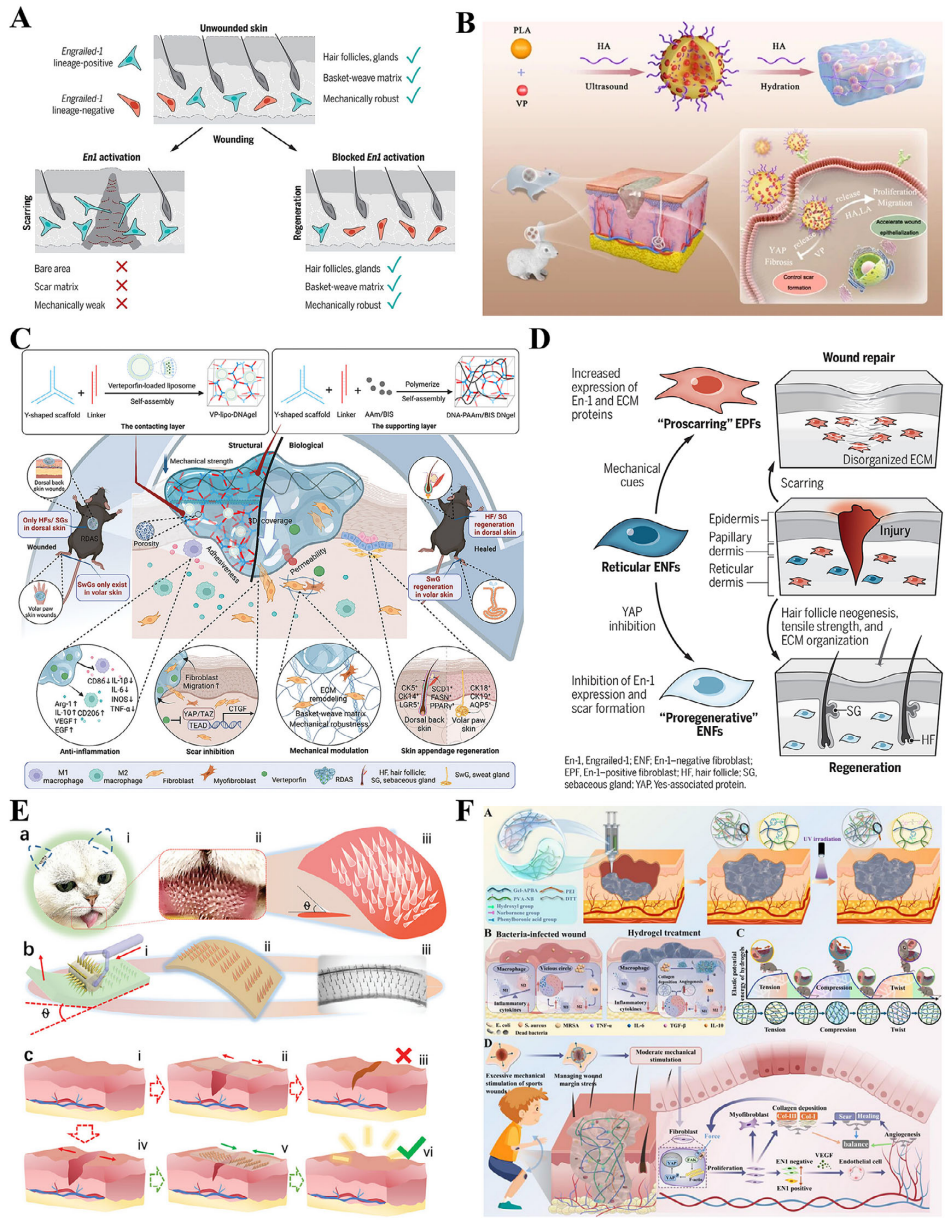
Regulating the fate of fibroblasts to guide skin regeneration. (A) Engrailed‐1 activation in skin fibroblasts drives scarring, and its suppression is a key target for achieving regenerative healing with appendage formation. Reproduced with permission [251]. Copyright 2021, American Association for the Advancement of Science. (B) Verteporfin‐loaded nanogels achieve scarless wound healing by repressing YAP‐En1 signaling and restraining fibrotic development. Reproduced with permission [156]. Copyright 2023, Chen K, et al. (C) Scarless skin regeneration via an ECM‐Mimetic DNA gel with VP that blocks myofibroblast differentiation. Reproduced with permission [257]. Copyright 2024, John Wiley and Sons. (D) Coordination of tissue regeneration by specific fibroblasts: inhibition of YAP signaling shifts fibroblast fate from proscarring to proregenerative phenotype, promoting hair follicle neogenesis and functional healing. Reproduced with permission [261]. Copyright 2021, American Association for the Advancement of Science. (E) Bionic tunable‐angle microneedles reduce wound tension, establishing a mechanical milieu that supports scarless regeneration. Reproduced with permission [262]. Copyright 2025, John Wiley and Sons. (F) Hydrogel blocks exogenous mechanical stress to suppress the YAP‐En1 pro‐fibrotic axis, significantly reducing scarring and enabling high‐quality tissue repair. Reproduced with permission [263]. Copyright 2025Reproduced under terms of the CC‐BY license, Zhang J, et al., published by John Wiley and Sons.

Inflammatory cells indirectly coordinate the fibrotic process through the secretion of key mediators, whereas fibroblasts serve as the ultimate effector cells that directly execute and drive the initiation and progression of fibrosis [249, 250]. Studies have shown that by suppressing the expression of Engrailed‐1 (En1) in fibroblasts to block the fibrotic program, regenerative healing encompassing appendage structures such as hair follicles and glands can be achieved [62, 251]. This indicates that En1 is a key target for achieving scarless healing (Figure 11A). Although YAP serves as a primary upstream regulator of En1, En1 can reciprocally regulate the activity and function of YAP, forming a positive feedback loop that collaboratively drives the biological process of fibrosis [251, 252]. Therefore, numerous studies have manipulated the YAP pathway in fibroblasts to reduce scar formation [253]. As an example, Verteporfin (VP), a YAP inhibitor, was incorporated into hydrogels for wound healing therapy [156, 254, 255, 256]. This approach effectively repressed YAP expression and nuclear localization, and blocked En1 activation thus restraining fibrotic development and eventually attaining scar prevention effects (Figure 11B). Researchers have engineered a programmable DNA hydrogel‐based artificial skin that facilitates scarless wound healing alongside the regeneration of hair follicles, sebaceous glands, and sweat glands by integrating an ECM‐mimetic DNA gel with VP to block fibroblast‐to‐myofibroblast differentiation (Figure 11C) [257]. Thus, interrupting the fibrotic program of fibroblasts by targeting the YAP‐En1 signaling axis represents a crucial strategy for directing wound healing toward authentic scarless regeneration. Additionally, during wound healing, such physical microenvironment formed by high tension and substrate stiffness acts as a crucial factor influencing fibroblast behavior [258, 259]. Just as the abnormally soft, low‐stress skin of spiny mice prevents excessive differentiation and survival of myofibroblasts, thereby guiding tissue toward regeneration rather than fibrosis [98, 99]. External stress is transduced into intracellular signals through mechanotransduction, which inhibits the Hippo pathway and promotes YAP nuclear translocation, thereby initiating a pro‐fibrotic genetic program [154, 260]. In contrast, inhibition of YAP signaling interrupts mechanotransduction‐mediated signaling, shifting the fibroblast fate from proscarring fibroblast (EPF) to proregenerative fibroblast (ENF) phenotype (Figure 11D) [72, 261]. This intervention concurrently promotes tissue regeneration, hair follicle neogenesis, tensile strength, and physiological ECM organization. In response to the clinical problem of scar formation in high‐tension wounds, researchers designed tunable‐angle microneedles (TPMNs) based on the biomimetic principle of a cat's tongue [262]. This system actively mitigates tissue tension by providing robust dermal anchorage, thereby establishing a mechanical milieu that supports scarless regeneration (Figure 11E). Additionally, by rapidly absorbing peripheral stress through elastic deformation and storing it as potential energy within its network, the hydrogel effectively blocks exogenous mechanical stimulation [263]. This suppression prevents the mechanically driven activation of En1‐positive pro‐fibrotic fibroblasts, leading to a significant reduction in scarring and facilitating high‐quality tissue repair (Figure 11F). Thus, the active intervention in the wound's mechanical microenvironment using biomaterials (e.g., biomimetic microneedles and hydrogels) to block the mechano‐activated YAP‐En1 pro‐fibrotic axis constitutes a promising strategy for directing scarless tissue regeneration.

In summary, a promising strategy involves designing smart biomaterial reservoirs that control the release of cells or signals to modulate key cellular fates (e.g., stem cells, immune cells, and fibroblasts). While preclinical evidence suggests this approach can influence cellular dynamics to mitigate fibrosis, translating these findings to achieve perfect regeneration remains a key goal for future research.

### 4.3 Sequential Multi‐Target Intervention in Pathological Processes: Based on the Dynamic Process of Scarring

Scar prevention could be regarded as a relay race that requires precise spatiotemporal coordination. Traditional uniform static interventions, such as single‐factor drug administration or passive dressings, often fail because they cannot align with the wound's intricate healing timeline [264, 265]. Emerging tissue engineering materials are progressively evolving toward intelligent strategies featuring multiple targets and temporal regulation through programmable materials [266]. This enables coordinated modulation of multiple pathological processes, including inflammation, proliferation, and remodeling, to systematically guide tissues toward regeneration.

#### 4.3.1 Harnessing Multi‐Target Synergistic Effects for Enhanced Therapeutic Outcomes

**FIGURE 12:**
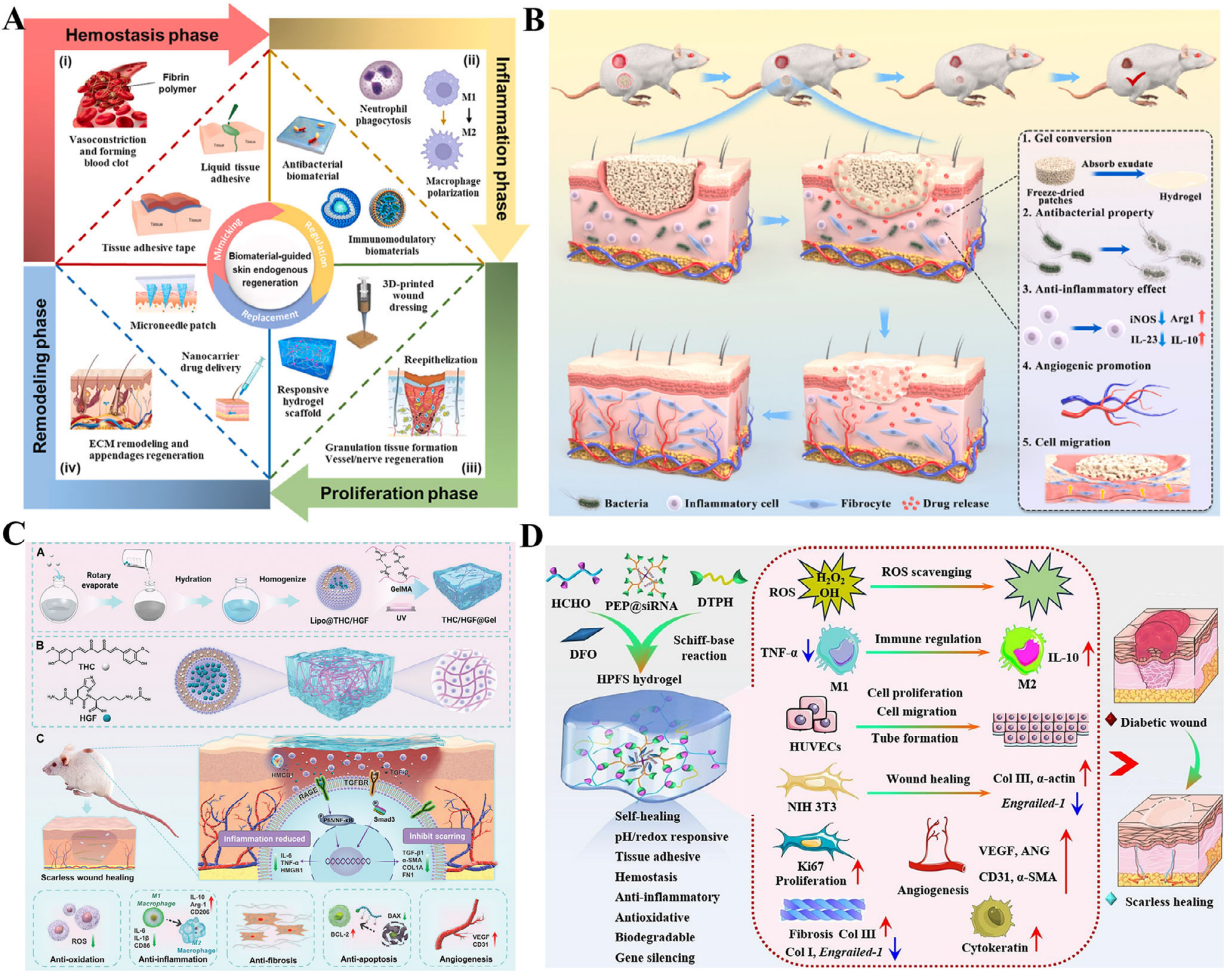
Harnessing multi‐target synergistic effects for enhanced therapeutic outcomes. (A) Multi‐target intervention strategy for wound healing stages, simultaneously modulating hemostasis, inflammation, angiogenesis, and ECM remodeling to block scar formation synergistically. Reproduced with permission [37]. Copyright 2024, Elsevier. (B) Bio‐patches convertible into hydrogels enable scarless wound healing via multi‐targeted antibacterial, anti‐inflammatory, and pro‐angiogenic effects. Reproduced with permission [271]. Copyright 2024, Ying X, et al., published by Elsevier. (C) Multifunctional nanocomposite hydrogel enables efficient scarless wound healing by multi‐targeted treatment. Reproduced with permission [250]. Copyright 2024, John Wiley and Sons. (D) Synergistic integration of En1 gene silencing with antioxidant, anti‐inflammatory, and pro‐angiogenic activities achieves scarless healing in diabetic wounds. Reproduced with permission [272]. Copyright 2025, Elsevier.

Traditional therapies that target a single factor often have limited effectiveness because the wound healing process involves multiple complementary mechanisms [267, 268]. Specifically, targeting a single pathway typically yields only transient and localized therapeutic effects, failing to block other persistently driven pathological processes such as infection, hypoxia, and excessive inflammation. In light of these limitations, the research model is progressively shifting toward multi‐target synergistic intervention strategies [221, 269]. This approach aims to more thoroughly disrupt the pathway of scar formation by generating additive or complementary synergistic effects and simultaneously modulating multiple stages of wound healing, including hemostasis, inflammation regulation, angiogenesis promotion, and ECM remodeling (Figure 12A) [37, 270]. Extensive studies have confirmed that such multi‐target systemic interventions exhibit significantly superior reparative outcomes in complex wound models compared to single‐target strategies, providing a critical pathway toward achieving high‐quality tissue regeneration [221]. Researchers have engineered a biomimetic mussel‐inspired bioadhesive using tannin‐cross‐linked citrate, which integrates antioxidant, anti‐inflammatory, and antibacterial functionalities, while demonstrating the ability to promote scarless wound healing [221]. In addition to the beneficial effects of antioxidant, anti‐inflammatory, and antibacterial activities on scarless wound healing, other researchers have designed a novel hydrogel composed of aloin, arginine, and alginate that accelerates the healing of acute infected skin wounds and demonstrates potential for scarless skin restoration in preclinical models through its antibacterial properties, anti‐inflammatory effects, and promotion of angiogenesis, cell migration, and differentiation (Figure 12B) [271]. Building upon conventional pro‐healing strategies, the research focus is increasingly shifting toward anti‐fibrotic approaches. A liposome‐composite hydrogel loaded with tetrahydrocurcumin and hepatocyte growth factor not only promotes fundamental wound healing through its antioxidant, anti‐inflammatory, and pro‐angiogenic functions but also proactively suppresses scar formation by inhibiting the TGF‐β/Smad signaling pathway, thereby achieving functional scarless regeneration of wounds (Figure 12C) [250]. Furthermore, leveraging the mechanism that suppressing En1 gene expression in fibroblasts blocks their fibrotic program, researchers have successfully achieved scarless healing in diabetic wounds by synergistically integrating En1 gene silencing with multidimensional modulation of the wound microenvironment, including antioxidant, anti‐inflammatory, and pro‐angiogenic activities (Figure 12D) [272]. Multi‐target scarless wound healing strategies are progressively shifting toward synergistic approaches that simultaneously regulate multiple pathological pathways, including infection, inflammation, hypoxia, and fibrosis. This direction is increasingly supported by preclinical evidence demonstrating that synergistic interventions significantly improve repair outcomes compared to single‐target strategies. While these synergistic interventions hold promise for advancing scar‐free tissue regeneration, their consistent translation into clinical applications remains a key challenge requiring further validation. Looking ahead, advanced bioengineering technologies may enable the development of fully personalized multi‐target therapeutic systems, with the long‐term goal of providing truly functional scar‐free regenerative solutions for diverse complex wounds.

#### 4.3.2 Achieving Spatiotemporal Precision for Stage‐Specific Intervention

**FIGURE 13:**
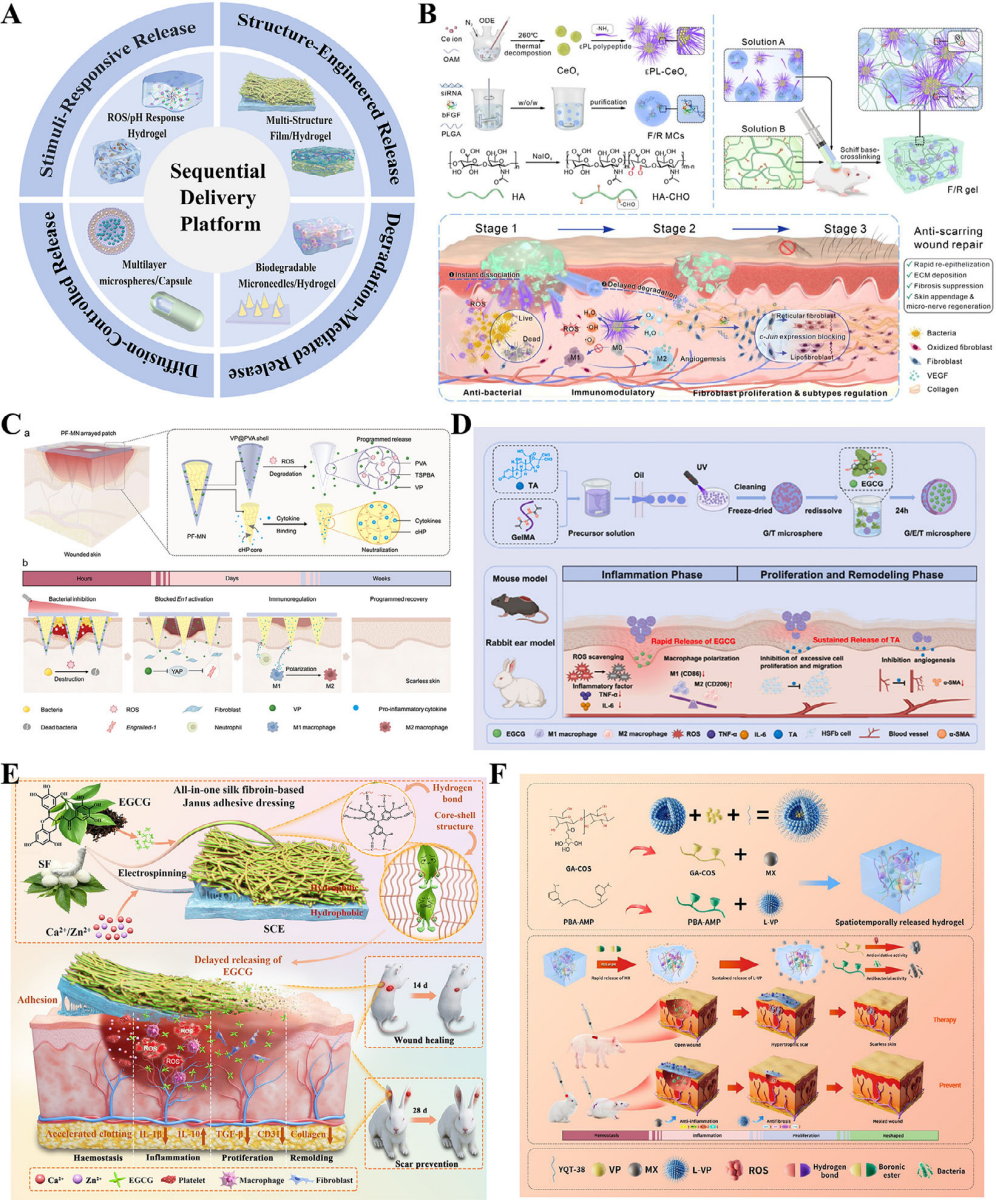
Achieving Spatiotemporal Precision for Stage‐Specific Intervention. (A) Sequential mimicking and modulating key structural and biological processes of wound healing to guide endogenous skin regeneration. (B) A dynamic Schiff base hydrogel enables scarless healing of infected wounds via a cascade‐responsive degradation mechanism for sequential release of therapeutic agents. Reproduced under terms of the CC‐BY license [264]. Copyright 2025, Zhang F, et al. (C) A programmable core‐shell microneedle patch achieves dynamic regulation of the wound immune microenvironment through sequential biofilm eradication, inflammation neutralization, and scar inhibition. Reproduced under terms of the CC‐BY license [254]. Copyright 2025, Zhang Y, et al. (D) A temporally adaptive multilayered GelMA microsphere system enables precise wound healing regulation through sequential and coordinated drug release. Reproduced with permission [265]. Copyright 2025, Luo D, et al., published by Elsevier. (E) A macro/micro dual‐scale designed Janus dressing achieves sequential drug release to regulate late‐stage angiogenesis and prevent scarring. Reproduced with permission [288]. Copyright 2025, Donghua University, Shanghai, China. (F) An environmentally responsive and programmable hydrogel enables scarless regeneration through temporal regulation of immune responses and fibroblast activation. Reproduced with permission [285]. Copyright 2025, American Association for the Advancement of Science.

Scar formation is essentially a dynamic pathological process where the inflammation‐proliferation‐remodeling stages are interconnected and progressively evolve [273, 274]. Therefore, the sequential modulation strategy, which dynamically aligns with the healing process, has become an important pathway for advancing the development of scarless wound dressings [21, 37]. This strategy employs advanced materials to achieve sequential coordination of multiple functions [264, 275]. Its primary objective is to precisely halt the fibrotic cascade at each critical phase of wound healing, thereby systematically enhancing the quality of tissue regeneration and ultimately achieving effective scar prevention [276]. Although scar prevention is a consensus goal in the sequential regulation of wound healing, the technological pathways for its implementation are highly diversified. A variety of drug delivery platforms with temporal control have been developed, primarily including degradation‐mediated, diffusion‐controlled, structure‐engineered, and stimulus‐responsive release systems (Figure 13A) [277, 278, 279]. Degradable biomaterials gradually release their encapsulated drugs during the degradation process, enabling drug concentrations to naturally increase or decrease according to the material's degradation rate, thereby ensuring biological signals exert maximal effects within the correct therapeutic time window [264, 280]. The researchers designed a dynamic Schiff base hydrogel that achieves precise treatment through a cascade‐responsive degradation mechanism [264]. It first rapidly releases antibacterial and anti‐inflammatory components to eliminate infection and remodel the microenvironment, followed by sustained delivery of pro‐regenerative factors and fibrosis‐inhibiting signals, thereby synergistically preventing scar formation and ultimately achieving scarless infected wound healing (Figure 13B). The multi‐scale design of materials at the macroscopic level represents a viable and effective strategy for achieving the temporal release of therapeutic agents [281]. For instance, core‐shell structures enable temporal control over drug release based on the diffusion behavior of the encapsulated substance [52, 282]. This facilitates the provision of precise, gradated bio‐signals at distinct stages of wound healing. Researchers have developed a programmable core‐shell structured microneedle patch that sequentially performs biofilm eradication, inflammation neutralization, and scar formation inhibition through the synergistic three‐step action of laser activation, shell degradation, and drug release, thereby achieving dynamic temporal regulation of the wound immune microenvironment (Figure 13C) [254]. From a structural design perspective, a bilayer microsphere system can be regarded as a core‐shell structure. Researchers have utilized microsphere carriers to achieve delayed release of TGF‐β inhibitors, enabling the provision of high drug concentrations during the critical window for scar formation inhibition, thereby effectively preventing scar generation [52]. Furthermore, by increasing the number of layers to form a multi‐layered microsphere system, more precise temporal control and functionally synergistic complex drug delivery strategies can be achieved [283]. Researchers discovered that a temporally adaptive GelMA microsphere delivery system, through precisely coordinated sequential release of epigallocatechin gallate and triamcinolone acetonide, enables comprehensive intervention in the wound healing process (Figure 13D) [265]. To achieve the sequential release of drugs within the body, the design of structure‐engineered materials is becoming key to breaking through this technological bottleneck [203, 284]. Based on a macro/micro dual‐scale design, the core‐shell Janus dressing was successfully fabricated. This dressing enables programmed release of (‐)‐Epigallocatechin‐3‐gallate (EGCG), allowing precise regulation of angiogenesis during the critical late stage of wound healing, ultimately achieving effective suppression of scar formation (Figure 13E). Additionally, the current research frontier is focused on developing intelligent material systems capable of dynamically responding to changes in the wound microenvironment and releasing active instructions at multiple targets according to a predetermined timeline [266]. Researchers developed a hydrogel possessing both environmental responsiveness and programmable drug release capabilities [285]. Through its dual functions of immediate inflammation suppression and continuous blockade of the YAP‐TEAD mechanosignaling pathway, this hydrogel achieves temporal regulation of immune responses and En1 fibroblast activation, thereby effectively promoting scarless wound regeneration (Figure 13F). The above studies collectively validate the potent efficacy of the sequential multi‐target regulation strategy for scarless wound healing. Its core principle lies in achieving the sequential delivery of active ingredients, thereby intelligently orchestrating therapeutic functions, including anti‐infection, anti‐inflammation, and regeneration promotion, and anti‐fibrosis, along the healing timeline [286]. This systematically coordinates pathological processes, achieving the transition of wound healing from passive repair to active regeneration.

In summary, scar prevention is shifting from single interventions to multi‐target temporal regulation, a transition increasingly supported by preclinical evidence demonstrating the feasibility of sequential strategies in modulating key pathological stages of wound healing. Researchers aim to precisely interrupt the fibrosis cascade at each critical temporal window through multi‐targeted, sequential intervention approaches. While these strategies demonstrate potential for reducing scar formation in preclinical models, their stable translation into clinical settings and the achievement of reliable scar minimization remain to be validated, representing future objectives that require fulfillment [287].

### 4.4 Integrating Emerging Technologies: Innovative Strategies Based on Smart Materials and Advanced Fabrication

Currently, cutting‐edge technologies are systematically advancing the development of next‐generation wound dressings, creating new opportunities for proactively promoting tissue regeneration and achieving scarless wound healing [289]. The field of wound healing dressings is witnessing continuous advancement, driven primarily by the integration of innovative smart materials with advanced manufacturing processes [181]. By integrating advanced technologies such as artificial intelligence, flexible electronics, and semiconductor technology, modern dressings can now sense changes in the wound microenvironment in real time and dynamically release therapeutic agents or deliver stimulation signals, which marks a shift from static coverage to dynamic management [290].

#### 4.4.1 Smart Materials Functional Evolution: From Static Coverage to Theragnostics Systems

**FIGURE 14:**
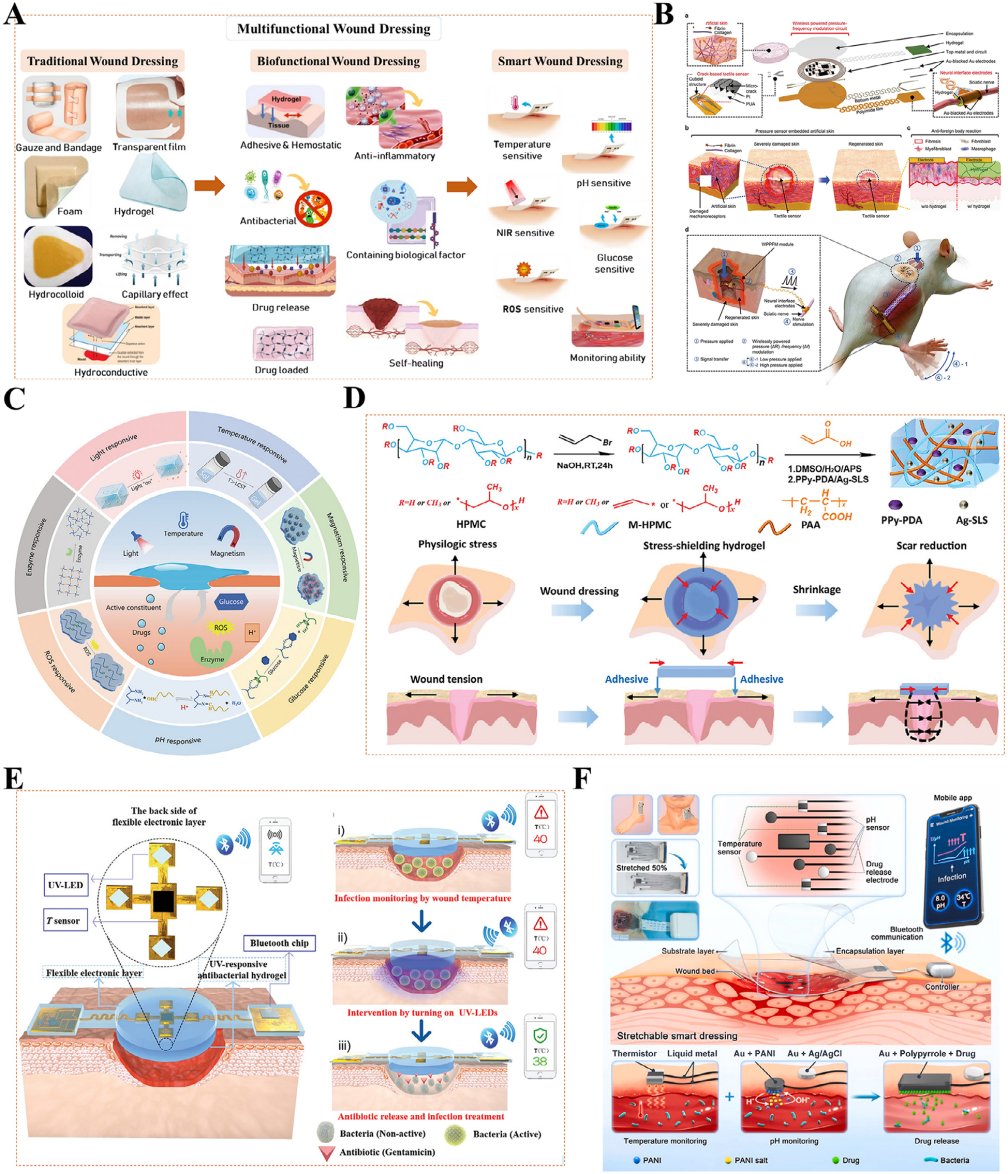
Smart materials functional evolution: from static coverage to active theragnostics systems. (A) Advancements in wound dressings: from traditional passive coverage to biofunctional and smart dressings with diagnostic capabilities. Reproduced under terms of the CC‐BY license [292]. Copyright 2025, Soylu Z, et al. (B) An implantable bioelectronic skin enabling sensory restoration and regenerative wound healing through synergy of tactile substitution and tissue regeneration. Reproduced under terms of the CC‐BY license [297]. Copyright 2024, Kang K, et al. (C) Signal sensing and dynamic response enable on‐demand intervention in wounds through real‐time detection and modulation of the microenvironment. Reproduced under terms of the CC‐BY license [298]. Copyright 2023, Chen Y, et al. (D) A body temperature‐responsive contractile hydrogel dressing for active remodeling of the wound mechanical microenvironment, thereby reducing scar formation. Reproduced under terms of the CC‐BY license [303]. Copyright 2024, Chen Q, et al., published by John Wiley and Sons. (E) A smart dressing with temperature monitoring, UV‐responsive drug release, and wireless communication for closed‐loop infection control. Reproduced under terms of the CC‐BY license [308]. Copyright 2024, Pang Q, et al., published by John Wiley and Sons. (F) A stretchable wound dressing detects bacterial infections via integrated pH/temperature sensors and enables on‐demand antibiotic release. Reproduced with permission [309]. Copyright 2024, Su R, et al., published by Elsevier.

Traditional dressings provide passive coverage, or further incorporate various biological functions such as antimicrobial properties, antioxidant effects, anti‐inflammatory properties, vascular regulation, conductive properties, and photothermal properties(Figure 14A) [221, 227, 269, 291, 292]. However, they still lack the ability to provide feedback on wound healing dynamics. To overcome this limitation, researchers have developed a phenolphthalein‐based hydrogel that exhibits a distinct color transition within the pH 5–9 range [293]. This allows for real‐time wound pH monitoring via smartphone imaging and machine learning‐based spectral analysis. This system represents a significant step forward in the visual monitoring of wound status, partially addressing the challenge of dynamic wound information feedback inherent in traditional dressings. However, it still does not possess the capability for active and intelligent regulation of the wound microenvironment. Currently, the focus of dressing research is shifting from providing static physical support toward constructing intelligent repair systems with sensing, decision‐making, and dynamic response functions, aiming to achieve precise and proactive intervention in the healing microenvironment [294]. Recent research is dedicated to developing smart dressings that integrate sensing and actuation capabilities to actively regulate the wound microenvironment [295, 296]. For example, researchers created a hybrid system enabling concurrent control of mechanical microenvironment, electric field, and hydrodynamics by coupling biomimetic adjustable microneedles with a triboelectric nanogenerator, consequently offering an innovative physical intervention methodology for scarless regeneration [262]. Moreover, other researchers designed a wirelessly powered implantable artificial skin with multiple functions including tactile substitution, tissue regeneration, and anti‐foreign body reaction, achieving the synergy between sensory recovery and wound repair (Figure 14B) [297]. Nevertheless, these systems remain inadequate in terms of initiative and adaptability when responding to dynamic wound signals and implementing closed‐loop control. To achieve active responses to dynamic wound signals and construct closed‐loop regulation systems, a series of intelligent materials capable of sensing the wound microenvironment and performing real‐time treatment have been developed [298, 299, 300]. These materials are equipped with signal perception and response capabilities, allowing them to dynamically modulate their functions through the detection of particular physical, chemical, or biochemical cues, thereby facilitating accurate on‐demand intervention (Figure 14C) [298, 301, 302]. Researchers developed a body temperature‐responsive contractile hydrogel dressing that achieves active remodeling of the wound mechanical microenvironment (Figure 14D) [303]. Additionally, researchers have developed a molecular sprinkler nucleic acid probe [304]. This probe can highly sensitively detect CTGF mRNA in scar fibroblasts and subsequently trigger the release of siRNA in situ, achieving simultaneous diagnosis and gene regulation therapy for pathological scars. Beyond responsive treatment, smart wound management is steadily moving toward diagnostic‐treatment integration, which is also known as theragnostics. Researchers have developed various smart dressings equipped with real‐time monitoring capabilities [182, 305, 306]. Their functions have evolved from simple visual detection of pH, temperature, electrophysiological signals, and biomarkers to integrated smart responsive materials that combine monitoring and intervention of the wound environment [307, 308, 309, 310, 311, 312, 313]. For example, some researchers have designed an integrated flexible electronic smart dressing system capable of temperature monitoring, UV‐responsive antibiotic release, and wireless communication, forming a closed‐loop management framework for infection warning, on‐demand treatment, and data transmission (Figure 14E) [308]. In addition, another study developed a battery‐free, wireless smart dressing based on near‐field communication and flexible electronics, which can simultaneously monitor temperature, pH, and uric acid levels while achieving on‐demand electro‐controlled antibiotic release, which offers a viable pathway for closed‐loop wound management [314]. Meanwhile, a study reported a stretchable wound dressing integrated with temperature and pH sensors. This device detects bacterial infections by monitoring these biomarkers and enables on‐demand antibiotic release through electrical control, which significantly improved the management of wound infection factors (Figure 14F) [309]. Although the aforementioned studies primarily focus on real‐time monitoring of the wound healing process, the successful progression of healing is a fundamental prerequisite for scarless repair. Therefore, these explorations in diagnosis‐treatment integration provide valuable references for the design of tissue engineering materials aimed at achieving scarless wound healing. Smart materials and advanced technologies are driving the evolution of wound management from standardized interventions toward personalized dynamic regulation. Although the integration of multiple technologies holds immense potential for developing full‐cycle, adaptive closed‐loop diagnostic and therapeutic systems, achieving scar‐free, perfect regeneration remains a long‐term goal. In fact, this approach faces significant translational challenges, including issues of biocompatibility, scalability, and clinical validation [315]. These challenges must be addressed to bridge the gap between technical feasibility and clinical application.

#### 4.4.2 Advanced Fabrication Technology Empowerment: From Design to Structure of Accurate Implementation

**FIGURE 15:**
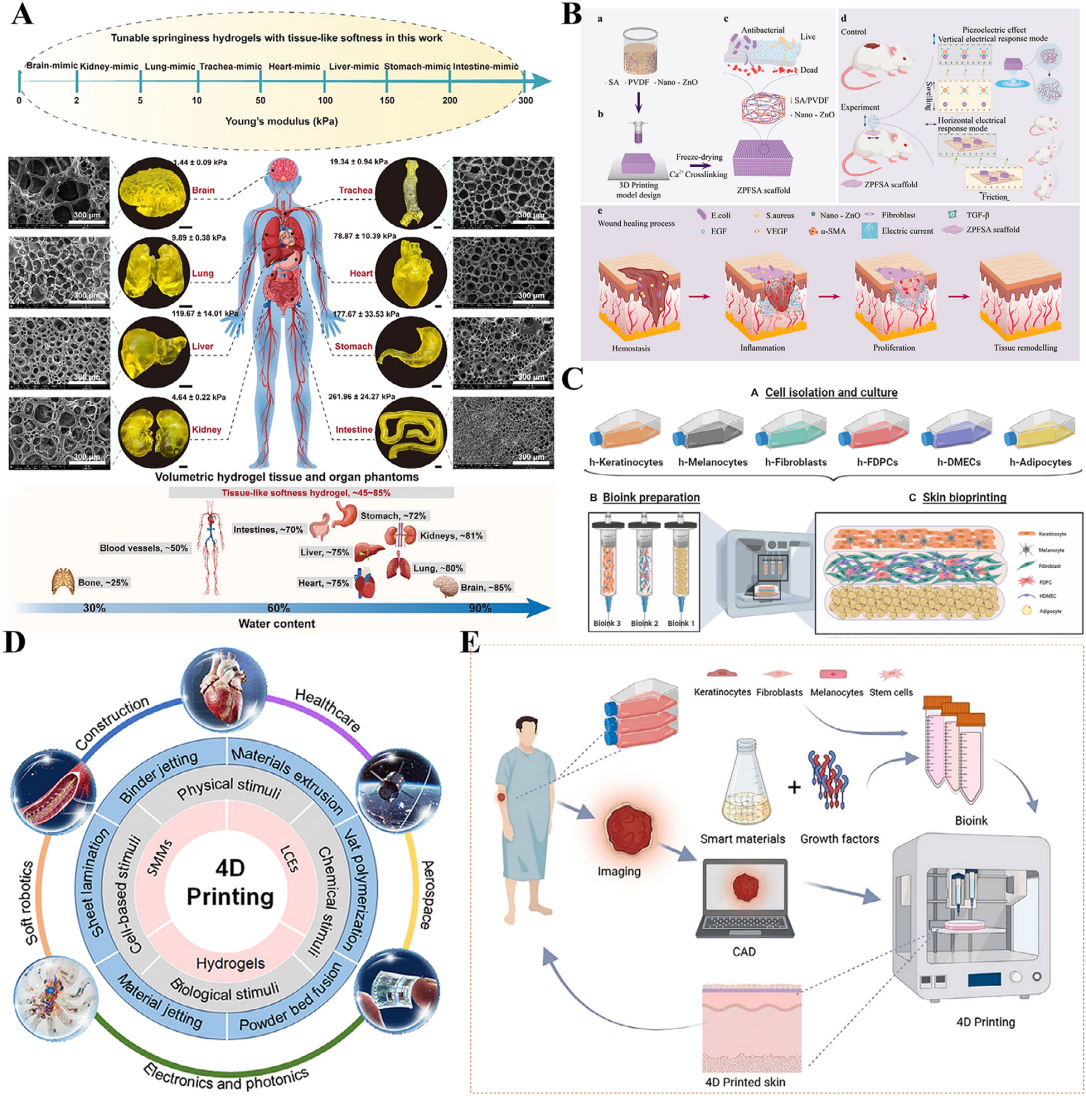
Advanced fabrication technology empowerment: from design to structure of accurate implementation. (A) 3D‐printed hydrogel volume‐based tissue and organ models with matched stiffness and bionic structures. Reproduced with permission [323]. Copyright 2023, John Wiley and Sons. (B) Integration of 3D printing and piezoelectric hydrogel for cascaded responsive wound therapy. Reproduced with permission [324]. Copyright 2022, American Chemical Society. (C) 3D‐printed trilayer biomimetic skin demonstrates exceptional restorative performance by enhancing vascularization and re‐epithelialization. Reproduced with permission [325]. Copyright 2023, American Association for the Advancement of Science. (D) Overview of 4D‐printed materials and structures with regard to printing techniques, stimuli, and applications. Reproduced with permission [339]. Copyright 2025, American Chemical Society. (E) Schematic representation of the 4D bioprinting workflow for personalized wound healing applications. Reproduced with permission [336]. Copyright 2025, John Wiley and Sons.

Additive manufacturing technologies are empowering the healthcare system at an unprecedented pace [316, 317, 318, 319, 320]. Traditional manufacturing methods (such as casting and molding) are incapable of producing complex, personalized internal structures, whereas current cutting‐edge additive manufacturing technologies can precisely translate the dynamic concepts of smart materials into physical entities with sophisticated functionalities [321]. 3D printing enables rapid and precise fabrication of customized scaffolds with complex structures. These scaffolds demonstrate significant potential in guiding tissue regeneration and modulating the wound microenvironment, offering innovative solutions for efficient healing [322]. Using tunable‐stiffness hydrogels and digital light processing 3D printing technology, researchers can now create soft tissue organ models, such as those of the brain and heart, that feature high‐fidelity complex structures, internal channels, and vascular networks [323]. These models show broad application potential in surgical training, device testing, organ‐on‐a‐chip systems, and other related fields (Figure 15A). In the preparation of wound dressings, using 3D printing technology, researchers have developed a PVDF piezoelectric hydrogel dressing that generates stable electrical currents by maintaining a moist environment combined with vertical swelling and horizontal friction triggered by exudate absorption [324]. This self‐produced current serves as exogenous electrical stimulation to accelerate wound healing, thereby achieving cascaded responsive therapy (Figure 15B). Additionally, researchers have successfully fabricated a three‐layer biomimetic skin containing epidermis, dermis, and subcutaneous tissue [325]. This functional full‐thickness skin replacement has shown exceptional restorative performance upon grafting, facilitating swift vascularization and enhanced re‐epithelialization to effectively suppress tissue contraction and fibrotic responses (Figure 15C). However, advancing 3D bioprinting technology into clinical applications necessitates addressing a core challenge: balancing biocompatibility with process compatibility [326]. On one hand, the printing process demands that bioinks possess suitable rheological properties, such as shear thinning to facilitate extrusion and rapid gelation to maintain structural fidelity, along with sufficient mechanical strength to withstand printing stresses and the stacking of multi‐layered structures [327]. On the other hand, once implanted in vivo, an ideal bioink should exhibit excellent biocompatibility (non‐cytotoxicity, low immunogenicity), support cell survival, proliferation, and functional differentiation, and degrade at a rate matching the ingrowth of new tissue [328, 329]. This prevents structural collapse from premature degradation or foreign body reactions from delayed residual material. To reconcile this tension, current research primarily explores solutions along two dimensions. The first approach encompasses composite modification strategies for bioinks, which synergistically integrate engineering support components and biological functional components [192, 328]. For instance, materials that afford transient printing stability and mechanical support, such as alginate and gelatin, are employed as the base matrix, and combined with highly bioactive constituents including hyaluronic acid and decellularized extracellular matrix (dECM) [208, 330]. This approach ensures printability while maximizing the regulatory role of biomaterials in the regeneration process. Second is the precise control of cross‐linking processes [331]. This includes optimizing photopolymerization efficiency with low‐toxicity, cytocompatible photo‐initiators, utilizing physical cross‐linking methods (e.g., thermosensitive and ionic cross‐linking), and constructing dual physical–chemical cross‐linking networks [332]. These strategies reduce the risk of cellular damage caused by chemical cross‐linkers while maintaining the scaffolds’ long‐term structural stability [333]. Via these multidimensional optimizations, a synergistic improvement in printability, biocompatibility, and regenerative functionality is finally realized. Building upon this foundation, bioactive 3D scaffolds and responsive delivery systems are developed to precisely modulate immune and growth signals on demand across distinct wound healing phases, thereby enabling sequential, multi‐targeted intervention against pathological processes [334]. Therefore, the clinical translation of 3D‐bioprinted skin tissue relies not only on improvements in printing precision and speed but critically depends on breakthroughs at the material level, achieving a perfect balance between manufacturing demands and biological requirements.

Building upon 3D printing, the more forward‐looking 4D technology introduces a temporal dimension [335, 336]. Its core lies in utilizing stimulus‐responsive materials such as shape‐memory polymers and smart bioinks to construct flexible structures capable of undergoing morphological or functional evolution post‐printing according to preset conditions (e.g., physiological environments), thereby opening new opportunities for biomedical applications (Figure 15D) [183, 337, 338, 339]. 4D bioprinting offers an innovative solution for personalized wound healing. This technology utilizes scanning and modeling combined with bioinks containing patient‐specific cells to create skin constructs with environmental responsiveness [336]. After implantation, the structure can adapt to the mechanical microenvironment of the wound, achieving active fitting and repair of the wound site through intelligent shape transformation, ultimately enabling efficient tissue regeneration and healing (Figure 15E). However, the advantages of 4D bioprinting in wound healing and skin tissue engineering remain underexplored, due to challenges including conceptual novelty, technical barriers, and ink selection [201]. Nevertheless, 4D bioprinting marks the transition of tissue engineering from static fabrication to dynamic biomimetics [339, 340]. With its unique spatiotemporal control capabilities, it holds promises for simulating the regenerative processes of scarless models and opens new pathways for achieving flawless scarless regeneration [341].

Although smart materials and advanced manufacturing technologies present revolutionary opportunities for scarless regenerative tissue engineering materials, translating these innovations from the laboratory to clinical applications remains fraught with multidimensional challenges [342, 343]. Posing a balance must be struck between technological innovation and practical feasibility. First, scaling up production and controlling costs represent the primary barriers to commercializing smart dressings [344]. Currently, manufacturing most smart systems (e.g., dressings integrating flexible electronics) and complex structures (e.g., high‐precision 3D/4D printed scaffolds) heavily rely on intricate laboratory processes, making it difficult to achieve low‐cost, high‐throughput, and batch‐to‐batch consistent mass production [345]. Additionally, batch stability of bioinks, consistency of printing parameters, and reproducibility of equipment are also critical factors limiting their clinical application [346, 347]. Future efforts must focus on developing low‐cost, scalable fabrication processes and establishing unified quality control standards to ensure product stability and reliability. Second, the complexity and uncertainty of regulatory approval pathways constitute a major bottleneck [346]. The regulatory classification of diagnostic‐therapeutic integrated smart systems incorporating sensing, feedback, and therapeutic functions remains ambiguous, potentially spanning medical devices, pharmaceuticals, and even biological products [348]. This ambiguity is particularly pronounced for bioelectronic dressings and 4D printed constructs loaded with viable cells or genetic materials [349]. Currently, approval standards for smart responsive dressings, closed‐loop feedback systems, and 3D bioprinted tissues remain under development. Developers must incorporate regulatory considerations early in research and development, which significantly increases the time cost and technical barriers to translation. Therefore, strengthening communication among academia, industry, and regulators to establish specialized regulatory pathways for smart regenerative medicine products is crucial for accelerating clinical translation. Finally, clinical adaptability cannot be overlooked. Many current cutting‐edge studies are validated in idealized animal models (e.g., mice), whose wound environments, dimensions, and healing dynamics differ significantly from complex human chronic wounds or extensive trauma [350, 351]. Future research must focus on more clinically relevant large animal models to systematically the long‐term safety, reliability, and therapeutic efficacy of such products, reliability, and therapeutic efficacy [256, 352]. Additionally, practical challenges such as data interpretation for smart dressings, clinical decision support, and seamless integration with existing medical workflows must be resolved before real‐world implementation. Thus, the clinical translation of smart materials and advanced manufacturing technologies relies not only on technical breakthroughs but also on coordinated advancement in scalable production, regulatory framework development, and clinical applicability. Through interdisciplinary collaboration and industry synergy, it is expected that these obstacles can be progressively overcome, propelling scarless regenerative therapies from the laboratory to clinical application.

In summary, this chapter systematically explores strategies for translating the biological blueprint of scarless regeneration into engineered reality, including bionic microenvironment fabrication, cell fate regulation, sequential multi‐targeted interventions, and technological integration. It is crucial to emphasize that the core rationale behind examining diverse biomaterial platforms, such as hydrogels, 3D printed scaffolds, and smart responsive systems, lies in unlocking their unique value as carriers and mediators for regulating regenerative mechanisms. These materials are not merely interchangeable nor hierarchical in terms of performance, rather they are selected and engineered based on their compatibility with specific regenerative mechanisms (such as immune regulation, ECM mechanical signaling, and developmental program reactivation) and the precision of their interventions. For instance, hydrogels excel at mimicking the physicochemical microenvironment of the ECM [35], smart materials achieve breakthroughs in the spatiotemporal dynamic regulation of regenerative signals [343], while 3D/4D printing and bioelectronic technologies aim to reconstruct multiscale structural and functional dialogues within tissues [336]. Therefore, evaluating the future development of these strategies should not be confined to comparing the physicochemical properties of the materials themselves, instead the focus should be on the depth of biological efficacy achieved and the clarity of the clinical translation pathway. Within this evaluation framework, it is necessary to objectively examine the current technical limitations of the concepts of “programmable” and “dynamic” regulation. Currently, most cutting‐edge strategies demonstrate significant potential in the precision of mechanistic intervention, particularly through stage‐adaptive approaches like microenvironment‐responsive release, achieving preliminary preclinical applications of dynamic regulation. However, truly adaptive in vivo feedback regulation (e.g., closed‐loop intelligent systems) still faces technical bottlenecks. Their successful translation requires further addressing core challenges including precise biological signal recognition, matched response speed, and long‐term biological safety. At present, the technical maturity, long‐term safety, and manufacturing scalability of these strategies remain critical hurdles to clinical adoption. Future breakthroughs will depend on deeper integration of materials science, developmental biology, and clinical medicine to create biocompatible engineered systems that combine intelligent regulation with the ability to safely and reliably execute complex regenerative programs. This will ultimately achieve a fundamental leap from passive repair to active regeneration.

## 5 Conclusions and Outlook

The healing of skin wounds is a sophisticated biological phenomenon governed by precise regulatory mechanisms, encompassing dynamically balanced processes such as inflammatory phase, proliferative stage, and tissue remodeling. This article systematically elaborates the wound healing process and factors influencing scar formation. Notably, natural scarless regeneration models, including early human fetuses and African spiny mice, offer crucial clues for understanding perfect tissue regeneration. However, the current understanding of scarless regeneration models (e.g., early‐gestation fetuses, African spiny mice) remains at the stage of mechanistic exploration. Future research should leverage cutting‐edge technologies such as single‐cell sequencing and spatial transcriptomics to systematically decipher the highly coordinated molecular networks underlying this process, thereby providing novel targets and theoretical frameworks for regenerative medicine [253, 353]. It is crucial to recognize that scarless healing is not the isolated effect of a single mechanism, but rather the result of spatiotemporally precise coordination among immune microenvironment regulation, ECM remodeling, and cell behavior guidance throughout the entire healing cycle. These three elements form an ordered cascade program that directly determines healing outcomes. Among them, the programmed regulation of the immune microenvironment during the inflammatory phase is a critical initiating factor [354]. The proliferative phase requires coordinated regulation of cell behavior and ECM dynamics, while the metabolic balance of the ECM during the remodeling phase directly influences tissue functional recovery [62]. Therefore, achieving scar‐free regeneration hinges on adhering to intrinsic temporal logic. By facilitating cross‐process dialogues among the immune microenvironment, ECM, and cells, precise interventions at key regulatory nodes, such as macrophage phenotypic switching, Hippo‐YAP signaling, and TGF‐β1/3 balance, can redirect the healing program from uncontrolled fibrotic repair toward ordered and complete regeneration. In‐depth analysis of this dynamic regulatory network will provide regenerative medicine with novel theoretical frameworks and therapeutic strategies that transcend single‐target approaches. Furthermore, to achieve a successful transition from regenerative models to clinical practice, we must not only understand the underlying molecular and cellular dynamics but also clearly recognize that the inherent complexity of the human body and stringent clinical safety requirements pose a unique barrier to this translation. Despite promising prospects, translating animal regenerative capacities to humans faces significant challenges. Human biological systems are far more complex than those of animal models, and ethical concerns surrounding stem cell research and cross‐species tissue engineering remain highly contentious. Successfully recapitulating these regenerative processes in humans necessitates addressing hurdles including immune rejection and long‐term safety risks. To achieve such recapitulation in humans, we must translate our deep understanding of scarless regeneration biological blueprints into engineering solutions capable of adapting to the complex human microenvironment. Therefore, the next phase of regenerative medicine will involve engineering innovations grounded in profound biological insights, characterized by high biological intelligence and clinical tolerability.

Current clinical measures for preventing scar formation, while achieving certain therapeutic efficacy, often fail to attain genuine structural and functional regeneration. Strategies for achieving scarless healing are undergoing a fundamental paradigm shift from passive anti‐fibrotic approaches to active, programmable pro‐regenerative strategies (Table 4). However, such approaches lack active modulation of the tissue's intrinsic regenerative potential, often resulting in scarless but non‐regenerative outcomes, where fibrosis is alleviated yet structural and functional integrity (e.g., skin appendage regeneration, mechanical elasticity recovery) is not restored. In contrast, proactive and programmable pro‐regenerative strategies adopt a regeneration‐centric paradigm, mimicking the spatiotemporal coordination mechanisms of natural scarless regeneration models (e.g., fetal skin, African spiny mice). It leverages smart materials and advanced manufacturing technologies to fabricate. By synergistically and stage‐specifically regulating immunomodulation, ECM remodeling, cell fate regulation, and signaling pathway balance, active strategies not only suppress fibrosis but also achieve de novo functional tissue reconstruction, thereby overcoming the fundamental limitations of passive approaches. In engineering biomimetic microenvironments, efforts should shift from biomimetic simulation to dynamic reconstruction, transcending static imitation of the composition and structure of the native ECM to focus on creating bioactive matrices with spatiotemporal evolutionary capabilities. In regulating cell fate, the efforts could evolve from broad‐spectrum regulation to precise dialogue, advancing toward high‐precision targeted communication at the cellular level to achieve ultimate control over cell fate. In sequential intervention in pathological processes, current research can further explore a shift from fixed regimens toward individualized adaptation. This approach would no longer rely on static material designs but would dynamically adjust therapeutic processes in response to individual healing progression, enabling multi‐target dynamic regulation of the wound healing cascade. In integrating emerging technologies, efforts can continue to strengthen the convergence of smart materials, flexible electronics, and bio‐manufacturing technologies. This will foster the development of diagnostic‐therapeutic integrated systems that combine monitoring, feedback, and treatment functions, achieving seamless integration of materials, drugs, devices, and data.

**TABLE 4:** Key differences between passive anti‐fibrosis and active programmable pro‐regeneration strategies.

Category	Passive anti‐fibrosis	Proactive and programmable pro‐regenerative
Core objective	Inhibit pathological fibrosis: Focusing on suppressing excessive collagen deposition and preventing hypertrophic scar formation to alleviate fibrotic outcomes.	Direct physiological regeneration: Guiding the wound healing cascade toward scarless, functional tissue reconstruction and promoting the regeneration of intact, fully functional tissues and appendages.
Action mechanism	Passively suppresses fibrotic signaling pathways and hyperactive cellular functions, with no active modulation of the native regenerative cascade.	Dynamically and programmably coordinates immune regulation, ECM remodeling, and cell fate regulation in a spatiotemporal manner to promote regeneration.
Intervention timing and logic	Post‐fibrosis correction: Intervenes after fibrosis is triggered or established, with a fixed, single‐time intervention mode.	Early‐stage programmed guidance: Conducts material‐based programming at early healing stages to orchestrate the healing cascade, with phased signal delivery synchronized with inflammation‐proliferation‐remodeling.
Regulation of cell fate	Suppresses pathological cellular activity: Mainly inhibits the activation, proliferation, and function of myofibroblasts to limit fibrotic progression.	Guides physiological cell fate decisions: Activates endogenous stem/progenitor cells and directs their ordered differentiation, and reprograms fibroblasts to prevent pathological myofibroblast transition.
Modulation of the wound microenvironment	Passive adaptation: Materials act mainly as drug carriers or physical barriers, with limited capacity to actively remodel and regulate the wound microenvironment.	Active biomimetic dynamic microenvironment construction: Materials are designed to mimic native regenerative ECM and dynamically regulate the healing microenvironment.
Key mechanism focus	Single‐pathway targeting: Inhibiting TGF‐β signaling, blocking pathological collagen cross‐linking, or applying physical isolation for scar reduction.	Spatiotemporal regulatory network coordination: Targeting the transition nodes of inflammation→ proliferation→ remodeling to dynamically and synchronously regulate the entire healing cascade.
Representative materials and strategies	Silicone gel sheets, anti‐fibrotic drug‐loaded hydrogels/films, compression materials.	Mechanically adaptive/stimuli‐responsive biomimetic hydrogels, sequential release systems, 3D‐bioprinted scaffolds, dECM composites, smart integrated patches.
Clinical outcome	Cosmetic improvement: Improves scar appearance but cannot restore native skin function.	Functional and structural restoration: Restores skin elasticity, extensibility and appendages, achieving near‐healthy physiological function.
Key limitations	Fails to target the root causes of fibrosis, risk of incomplete wound healing, scar recurrence, and lack of functional tissue recovery.	Involves sophisticated engineering design, faces challenges in balancing biocompatibility, precise programmability, and translational clinical feasibility.

Research on novel biomaterials has demonstrated considerable potential in terms of structural design, functional properties, and composite architecture. However, a significant translational gap persists between laboratory achievements and clinical application. In clinical regenerative therapeutic systems, achieving clean adhesion to skin and wound surfaces is crucial for ensuring the long‐term stability of therapeutic agents [355]. The dynamic wet microenvironment of skin and wounds (e.g., exudate, sweat) combined with biological anti‐adhesion factors (e.g., protein contamination) can compromise the stability of therapeutic interfaces [356]. This undermines controlled drug release, scaffold fidelity, and sensing efficiency, posing a significant challenge for clinical translation. An ideal therapeutic interface must satisfy the requirements of stable adhesion‐biological safety‐functional compatibility, meaning it achieves long‐term, non‐damaging clean adhesion without interfering with microenvironment reconstruction and regenerative regulation processes. To address this challenge, cutting‐edge research is drawing inspiration from biomimicry and materials chemistry [357, 358]. For instance, multi‐scale suction‐driven architectures inspired by octopus suckers can overcome liquid barriers through negative pressure, enabling controllable adhesion from the nanoscale to macroscale [359]. Future efforts should integrate advanced adhesion technologies with core modules like microenvironment reconstruction, cell fate regulation, and sequential delivery. This will establish patient‐friendly regenerative therapeutic systems, achieving a high level of integration between engineering reliability and biological regenerative efficacy. The continuous innovation of intelligent bionic materials is central to the pursuit of the ultimate goal: achieving highly efficient and perfect skin regeneration. Ultimately, through interdisciplinary integration and the innovative application of cutting‐edge biotechnology, it is anticipated that we will achieve proactive guidance and precise regulation of the wound healing process. This will advance skin scar treatment beyond the current passive intervention model, which primarily improves appearance and symptoms, toward an active reconstruction model that promotes complete regeneration of both structure and function. The ultimate goal is to attain scarless healing in humans.

## Funding

This work was supported by the Natural Science Foundation of China (Grant Nos. 82272453, U21A2099, and 82472133), and the Guangdong Basic and Applied Basic Research Foundation (Grant No. 2024A1515012664).

## Conflicts of Interest

The authors declare no conflict of interest.

## Data Availability

The authors have nothing to report.
